# How level of understanding and type of used sources relate to adherence to COVID-19 public health measures in Canada

**DOI:** 10.1038/s41598-023-38824-0

**Published:** 2023-08-11

**Authors:** Clémentine Courdi, Sahar Ramazan Ali, Mathieu Pelletier-Dumas, Dietlind Stolle, Anna Dorfman, Jean-Marc Lina, Éric Lacourse, Roxane de la Sablonnière

**Affiliations:** 1 Department of Sociology, Université de Montréal, Montreal, Canada; 2 Department of Psychology, Université de Montréal, Montreal, Canada; 3 Department of Political Science, McGill University, Quebec, Canada; 4 Departement of Psychology, Bar Ilan University, Ramat Gan, Israel; 5 Departement of Electrical Engineering, École de Technologie Supérieure, Montreal, Canada

**Keywords:** Health policy, Human behaviour

## Abstract

Previous studies have highlighted the importance of promoting health literacy and minimizing misinformation to encourage higher adherence to key public health measures during the COVID-19 pandemic. This study explores how one’s self-reported understanding of information and types of sources used to get information regarding COVID-19 can hinder adherence to public health measures implemented by the Canadian government. Data was collected following a longitudinal design of 11 time points for April 2020 to April 2021. The sub-sample used for this study included 2659 Canadians who completed the survey for at least four time points. Using Latent Class Growth Analysis, we modelled typical trajectories of adherence to three key public health measures: staying home, social distancing and mask wearing. Overall, a lower level of understanding was associated with lower adherence trajectories to public health measures, and vice-versa. Adjusted odds ratio (AOR) showed that the higher the level of understanding, the higher were the chances of following a high adherence trajectory. The type of used sources also showed a significant statistical association with adherence trajectories for social distancing and staying home (AOR: between 1.1 and 3.4). These results are discussed considering future policy implications.

## Introduction

As the health crisis related to the COVID-19 pandemic unfolded, Canadian federal and provincial governments gradually implemented a variety of public health measures to limit the spread and impact of the COVID-19 virus. Understanding what can promote or limit adherence to public health measures is crucial to control the current and future health crises. One important aspect that emerged during the pandemic is the risk posed by misinformation. Misinformation about COVID-19 and related measures can occur due to the type of sources from which individuals obtain information, or through a simple lack of understanding regarding complex information. This study explores how individuals’ perceived level of understanding, and the type of sources (official or informal) individuals rely upon most regarding COVID-19 can hinder their adherence to the public health measures implemented by Canadian federal and provincial governments. Due to the evolving nature of the health crisis, it is important to evaluate adherence to public health measures in the long-term, thus considering changes in behaviors over time. Furthermore, there is a lack of prior research on the relation between adherence to public health measures and understanding information regarding these measures in the context of COVID-19. Thus, this study aims to examine adherence to public health measures throughout the first year of the COVID-19 pandemic by modeling latent trajectory groups of adherence to three key public health measures (staying home, social distancing and mask wearing) and considering how perceived understanding of information and type of used sources can be associated membership in these trajectory groups.

### Adherence to public health measures

From the very start of the COVID-19 pandemic, adherence to governmental guidelines was an essential component of crisis management. Adherence to public health measures in the event of global health emergencies is essential to control the evolution of the crisis, making it important to understand what can influence a population’s response to such measures^1^. During the first year of the pandemic, a considerable number of preventative public health measures were implemented to limit the spread of the COVID-19 virus. Hence, researchers quickly focused on identifying public health measures that were the most effective in diminishing transmission rates of the virus. Across different disciplines, from microbiology to psychology, three key public health measures were frequently identified in the literature: (1) maintaining social distancing in public, (2) staying home or avoiding gatherings and (3) wearing a mask in public spaces^2,3^. These measures were considered as the most influential to reduce cases of infections and like most public health measures, require a high level of adherence from the population to be efficient^4–6^. However, governments and scientists realized early on that these public health measures were not consistently and homogeneously followed in the Canadian population^7^. For example, a study in Quebec identified several patterns of adherence to physical distancing, distinguishing a high adherence trajectory, a slowly declining trajectory, an unstable adherence trajectory, and a rapidly declining trajectory^8^.

### Latent Class Growth Analysis during COVID-19

The COVID-19 pandemic lasted longer than expected, which implies revisiting phenomena such as changes in behavior from a longitudinal perspective. This is possible by conducting Latent Class Growth Analysis (LCGA) that aims to group individuals with similar response patterns across time^9,10^. Each trajectory can then take different shapes (constant, linear, quadratic or cubic). This type of analysis has already been applied in various fields during the pandemic. For example, a team of researchers in Canada evaluated changes over time in depressive symptoms among adult workers^11^. This research revealed that there are patterns of response that persist over time, allowing government agencies to formulate more targeted solutions. However, LCGA has not yet been applied in the context of public health measures, which would allow to identify adherence trajectories and the risk factors associated with them.

### Misinformation: an issue related to the type of used sources

Misinformation is typically defined as the presentation and diffusion of erroneous or misleading information^12,13^. However, it also designates the state of being misinformed, which can stem from the consumption of biased sources, but also from a lack of understanding or knowledge leading to misinterpretation or misunderstanding of information^13^. Through both those perspectives, misinformation quickly became a significant concern with the emergence of COVID-19^14,15^. Among many potential social, economic and cognitive factors related to adherence, misinformation was shown by numerous studies to be significantly associated with non-adherence to public health measures^16–18^. Indeed, misinformation due to the consumption of unverified sources regarding COVID-19 can hinder adherence to public health measures, and even have important consequences beyond lack of adherence, depending on the erroneous narrative to which individuals adhere^12,19,20^. In the context of COVID-19, most researchers distinguished official sources transmitting information in accordance with the most recent scientific findings and considering the best outcomes for the general population, in contrast to informal sources that could diffuse unsupported information regarding COVID-19^18–21^. For example, increase in fake news, conspiracy theory and general false information are a significant concern, because this type of misinformation tends to be associated with a lack of adherence, but also to behaviors that actively increase the risk of infection and transmission^14,22,23^. Moreover, the type of sources used by individuals modulates the level of adherence to public health measures: for example, citizens relying on government sources have a higher adherence level compared to those relying on mainstream media or on social media^18,19,24^. In addition, some authors have also pointed out that the types of sources favored by individuals when seeking information about COVID-19 are significantly related to the level of knowledge about COVID-19^13,14^. However, misinformation cannot be attributed solely to the type of used sources, as a general understanding of health measures is also essential for adherence to public health measures.

### Understanding information relative to COVID-19

It is widely accepted in health care that understanding information is critical to improving patient adherence to treatment and counter misinformation^25^. However, few studies have focused on this issue in the context of COVID-19, leaving a blind spot in public health strategies to promote adherence to public health measures^26,27^. Understanding information can also be integrated into the broader concept of health literacy. It refers to the ability of individuals to access, understand, and use information about their health, and can be measured either through an “objective” assessment of one’s knowledge or individuals’ perception of their own understanding^16,28,29^. In the case of COVID-19, public health measures (along with vaccination) represent the interventions that are sought to be adhered to by the general population. Of course, promoting understanding through better health literacy at the general population level is challenging. Despite that challenge, many authors insist on the fact that understanding information is critical to the success of public health interventions and the reduction of health inequalities^25–28^. Currently, health literacy is called to have an even more important role as it is being combined with media literacy to improve understanding of the disease, the associated public health measures and reduce misinformation^13,16,30^. Understanding information related to COVID-19 and to public health measures is also important for the proper application of public health measures: for example, understanding that COVID-19 is an airborne virus might encourage an individual to wear a mask and to keep distances from others^13,18^. Considering that public health measures are most efficient when a large portion of the population adheres to them, promoting a good understanding of public health measures is essential to increase adherence and ultimately lower transmission^12,29–31^. Misinformation does not stem only from the reliance on official or informal sources of information: the lack of understanding can be in itself a cause of misinformation and consequently, lower adherence to public health measures.

### Sociodemographic characteristics linked to adherence to public health measures

Several studies explored the association of various individual characteristics with adherence to public health measures. For example, it has been shown that younger individuals and men are less likely to adhere to public health measures^32–35^. Predictors such as lower education and lower socioeconomic status appear to follow the same trend of low adherence to health recommendations^4,35–37^. Region of residence in Canada is also an important predictor of adherence to public health measures. For example, one study reported that residents from Alberta were less keen in endorsing contacts’ limitation measures and were more hesitant about governmental guidelines than residents from Ontario^38^. In terms of political identity, the literature suggests that individuals who adopt a more conservative ideology tend to be less concerned about the consequences of the virus and less likely to adopt preventive measures^39,40^. Finally, some studies reported that non-immigrants were more likely to adhere to public health measures than individuals from migrant descendance^41,42^.

In this study, we aim to identify factors that are associated with lower adherence to three key public health measures (staying home, social distancing and mask wearing) in the long term. Considering results derived from previous studies, both regarding COVID-19 and similar instances, we can expect that people who perceive they don’t understand information regarding COVID-19 and public health measures are less likely to follow these measures in the long term. Moreover, it is expected that people who rely mostly on informal sources to get information regarding COVID-19 and public health measures are less likely to follow them in the long term. We also postulate that only a small proportion of the population will display a very low level of adherence throughout the pandemic.

## Methods

### Participants

This study is part of the project "COVID-19 Canada: The end of the world as we know it?". The longitudinal survey at the core of the project was conducted in collaboration with the polling firm *Delvinia* and uses a survey panel called *AskingCanadians* (inventory of over one million Canadians). Participants selected from this database, who were financially rewarded for their participation, had the option of completing the questionnaire on multiple interfaces such as a mobile phone, tablet, or computer. This longitudinal survey has twelve time points spread over 2 years, from April 2020 to April 2022. For this study, we only refer to the first eleven time points, spanning from April 2020 to April 2021. Time was coded by weeks to account for the different spacing between each time points (see Supplementary Materials, Table 1). A non-probabilistic sample of 3617 participants were accounted for at the first time point, and a weighted quota method was applied. Although the participants were selected to ensure representativeness according to Statistics Canada population data, the sample is less representative for certain demographics such as Francophones, Canadians with a low level of education and Aboriginal people. Due to attrition, the average participation rate for time points 2 to 12 was 55%. To limit the effect of non-representativeness, we incorporated weights by demographic characteristics (age, gender, province of residence, household income). We also integrated two statistical methods to limit the impact of missing data, such as "full information maximum likelihood" estimation and multiple imputation. For more details on the recruitment, data cleaning and weighting, refer to the technical reports of the project^43,44^.

For this study, only participants who completed at least four time points of the survey were considered, as that was necessary for the modelization of latent trajectories. Thus, all results shown in this article pertain to this sub-sample of 2629 participants. Since the data was weighted to be representative of the Canadian population in terms of age, gender, province of residence and education, no significant differences in terms of these sociodemographic characteristics were noted between this sub-sample and the original whole sample.

### Adherence to public health measures

**Figure 1:**
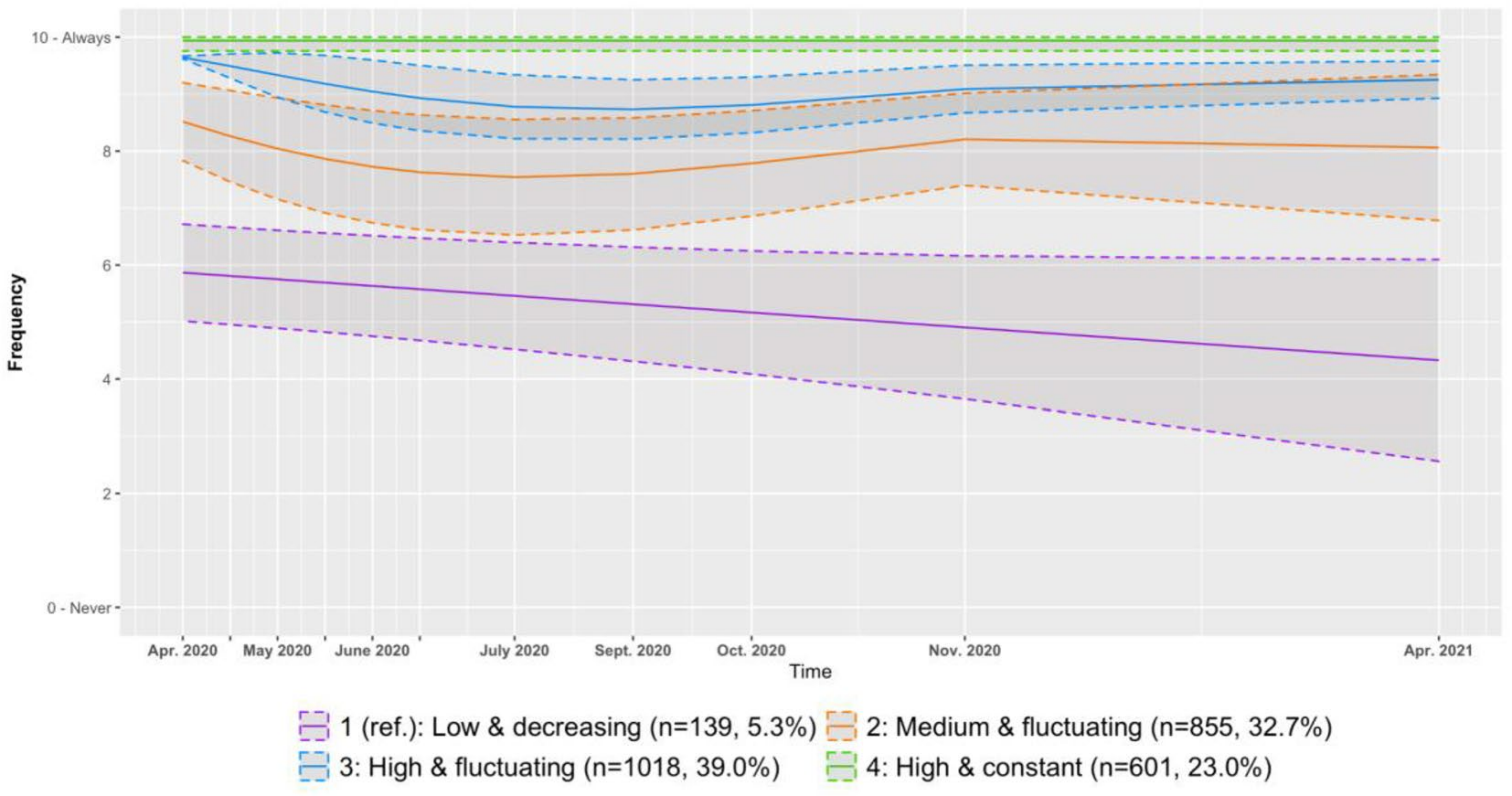
Trajectories of adherence to the social distancing measure with 95% confidence interval.

**Figure 2:**
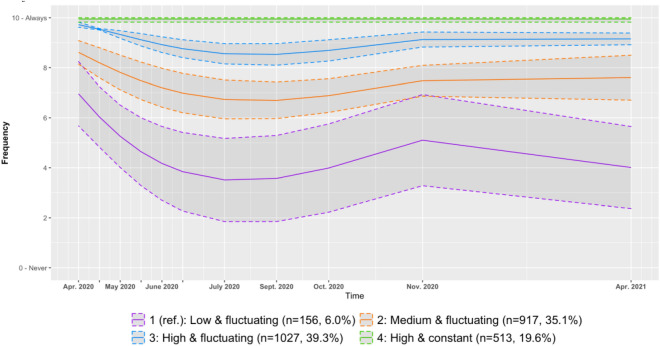
Trajectories of adherence to the contacts limitation measure with 95% confidence interval.

**Figure 3:**
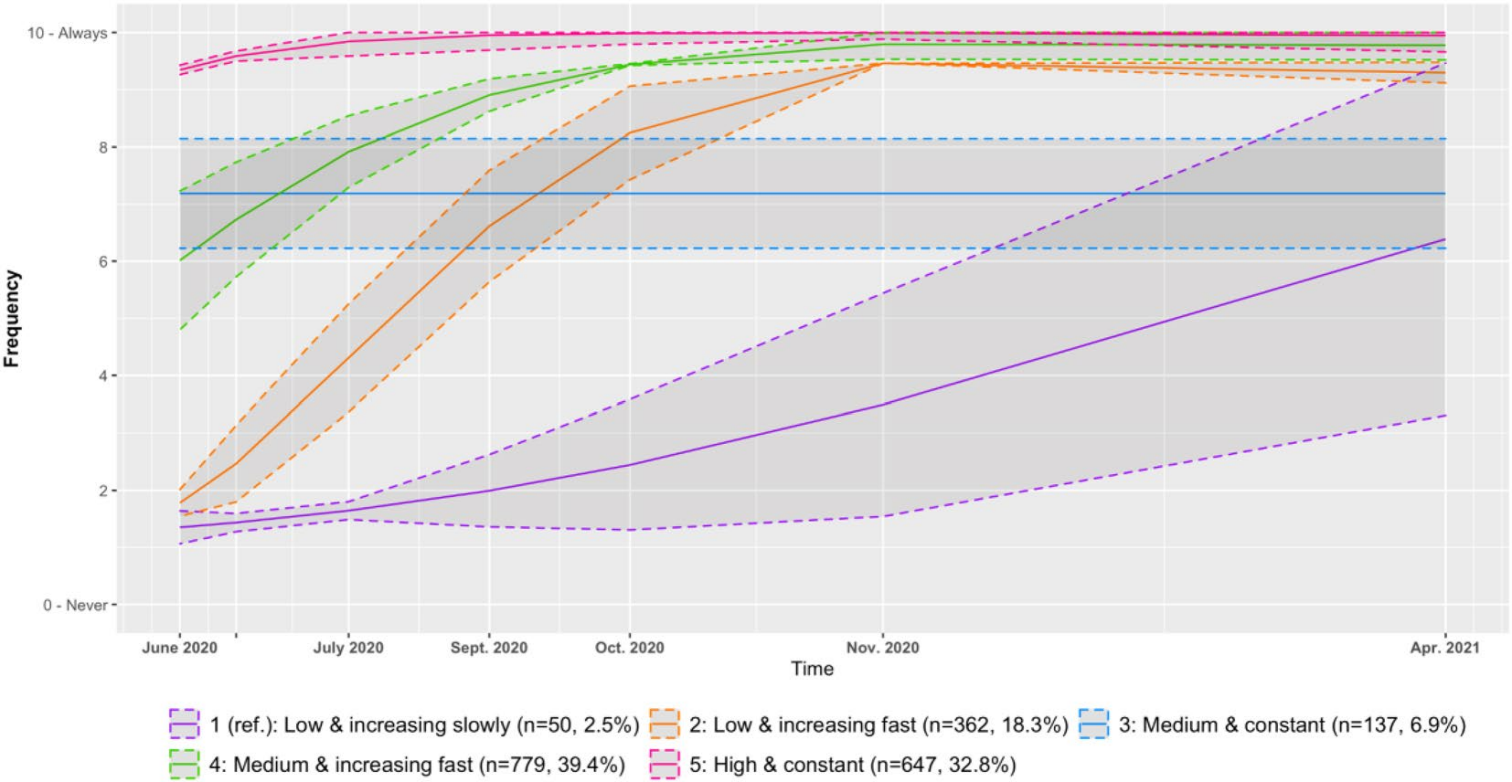
Trajectories of adherence to the mask wearing measure with 95% confidence interval.

Participants reported their level of adherence to the measures related to physical distancing and contacts limitation (staying home) at all eleven time points (from April 2020 to April 2021). Mask wearing was measured from the fifth time point (July 2020 to April 2021). The participants answered on a scale from 1 to 10 (never to always) regarding the following statements: "Currently, how often do you do the following? (1) Maintain a distance of at least two meters (about two arm's lengths) from others when I am not at home; (2) stay home as much as I can; (3) wear a mask in public". These measures were inspired from the *Institut national de santé publique du Québec* [National Institute of Public Health of Quebec]. Adherence to sanitary measures could also be operationalized as a single variable, but since this study’s objective was to elicit differences in adherence to each sanitary measure, they were each considered separately. Variables for each public health measure were strongly correlated between waves, but only moderately correlated between the three public health measures considered (see Supplementary Materials, Table 3, Figure 1, 2, 3).

### Perceived level of understanding

To evaluate the level of understanding (as self-reported by participants), we used two knowledge-related questions that were adapted from Zhong et al. ^45^ and Krägeloh et al. ^46^ in order to measure the participants' level of understanding. We assessed participants’ level of agreement on a 10-point scale (1 = Strongly *disagree*, 10 = *Strongly agree*) with two statements: (1) In general, I have a clear understanding of the various measures established by Canada’s public health agency; (2) In general, I have a clear understanding of the various measures established by my provincial public health agency. These two items were strongly correlated (r = 0.64). The Cronbach’s alpha is moderately strong (0.78 [0.74, 0.81]), which justifies the use of these two items to create the variable used in this study. It is important to keep in mind throughout the article and interpretation of results that the level of understanding refers to a self-assessment by participants of their own understanding, just as adherence is based on self-declared behaviours. Basing the measure of understanding on self-assessment is common when studying understanding of health information as a component of health literacy^16,26,29^.

### Type of sources used for information regarding COVID-19

**Figure 4:**
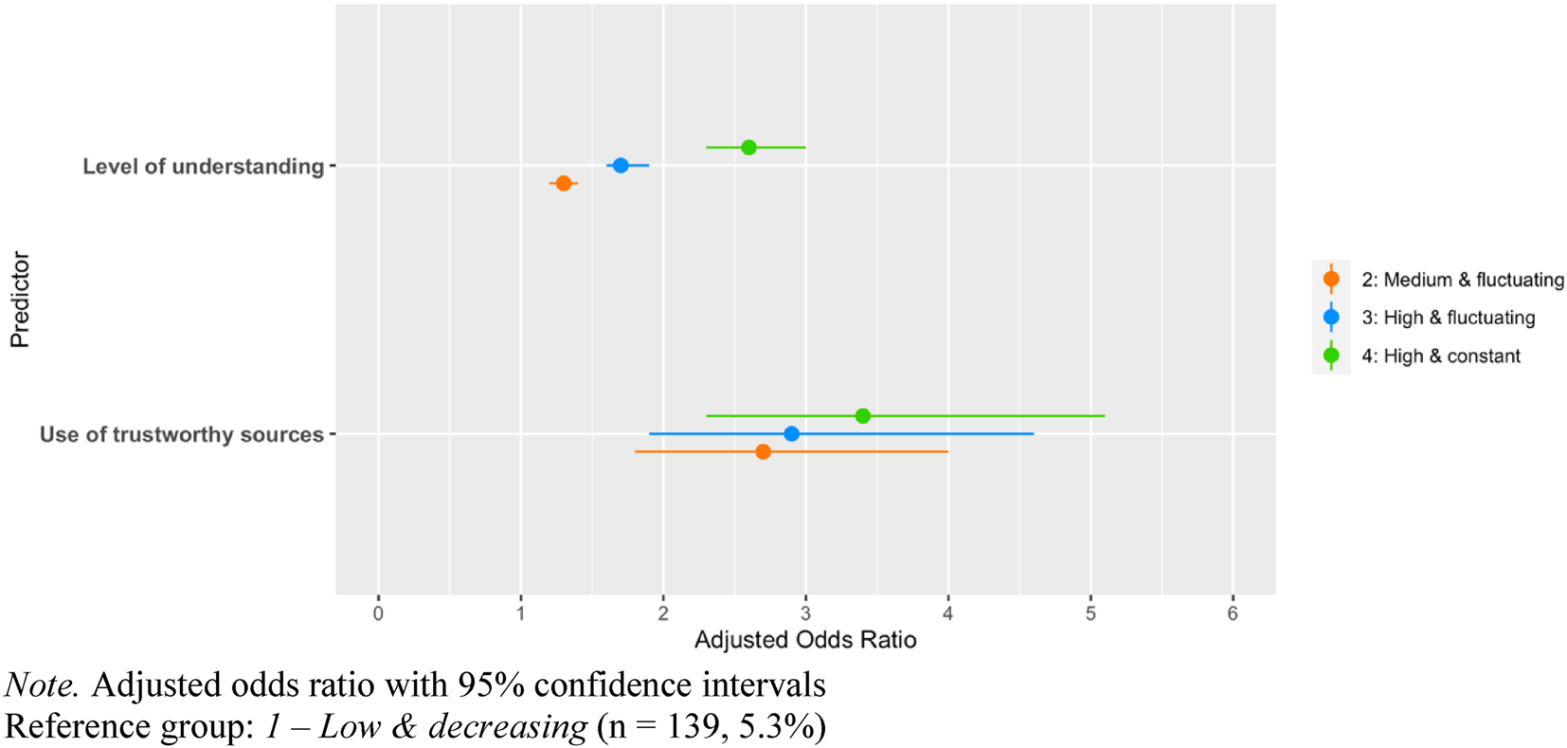
Predictors of adherence to social distancing. *Note* Adjusted odds ratio with 95% confidence intervals. Reference group: 1—Low and decreasing (n = 139, 5.3%).

**Figure 5:**
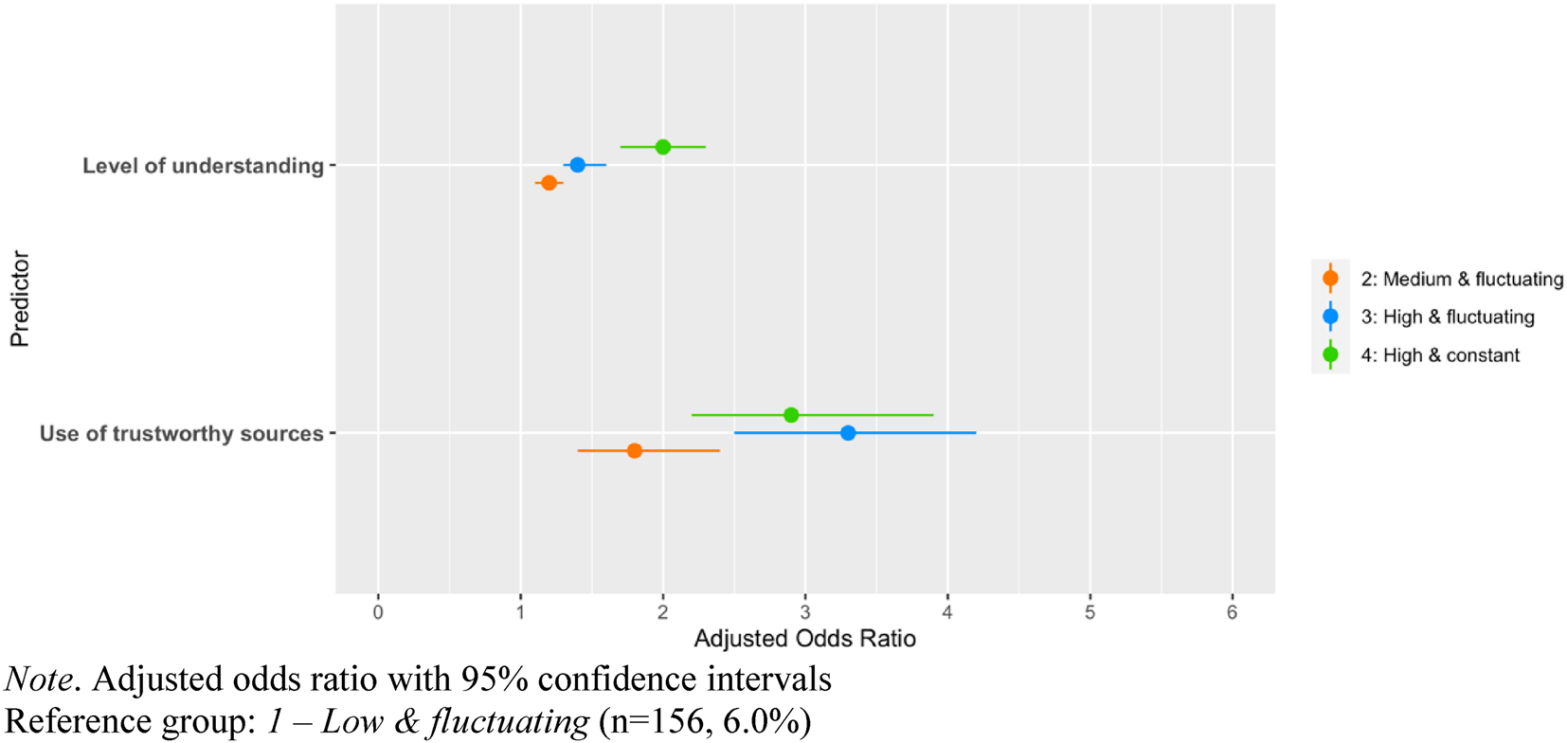
Predictors of adherence to contacts limitation. *Note* Adjusted odds ratio with 95% confidence intervals. Reference group: 1–Low and fluctuating (n = 156, 6.0%).

Regarding the sources of information, we asked participants the following question: "Which of the following sources do you trust the most when you want to be informed about COVID-19? Select up to three answers starting with the most trusted source. (1) family, friends, and colleagues; (2) workplace; (3) local/national/international news (newspapers, television, radio, online); (4) my doctor or healthcare professional; (5) provincial health authorities and provincial government; (6) my community/religious/cultural leaders; (7) The World Health Organization; (8) The scientific literature; (9) other people or groups via social media or the internet". The original version of this question was created by the Montreal Behavioural Medicine Center for the iCare Study (2021), an international research project aiming to assess and optimize strategies for “flattening the curve” of COVID-19 infections^47^. Based on this study’s results and our own preliminary analysis of correlations between sources and adherence, we divided sources into two categories: official sources (Local/national/international news (newspapers, television, radio, online); My doctor or healthcare professional; Provincial health authorities and provincial government; The World Health Organization; The scientific literature) and informal sources (Family, friends, and colleagues; Workplace; My community/religious/cultural leaders; Other people or groups via social media or the internet). Participants were then assigned the score of 0 if they used only official sources (n = 2331, 88.7%), and a score of 1 if they reported using at least one informal source (n = 298, 11.3%). More details for the construction of this variable can be found in the Supplementary Materials (Table 4, Figure 4, 5).

### Sociodemographic characteristics

**Figure 6:**
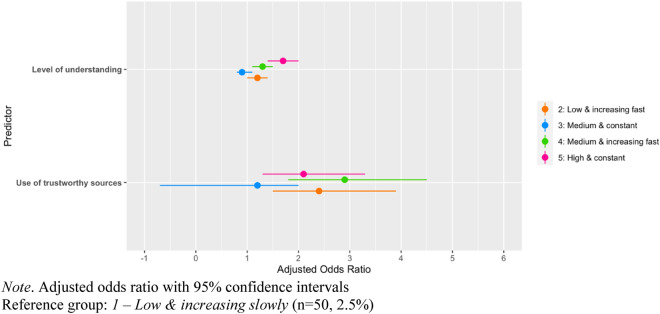
Predictors of adherence to mask wearing. *Note* Adjusted odds ratio with 95% confidence intervals. Reference group: 1—Low and increasing slowly (n = 50, 2.5%).

Many sociodemographic characteristics such as age or gender have a well-documented influence on adherence to public health measures. Thus, we included the six following factors as control variables in our analysis: age, gender, education level, political identity, region, and immigration status. Participants were asked the following question regarding their political identity: "Regarding politics, people often speak of the "left" and "right." Where would you place yourself on the following scale?". They were also questioned on their immigration status: "What is your country of birth". Political identity was evaluated on a scale of 1 to 10 (left to right), while country of birth was converted to a binomial (born inside or outside Canada). All covariates were uncorrelated between themselves (see Supplementary Materials, Table 5, Figure 6).

### Statistical analysis

In this study, we used LCGA, or also referred as Group-based trajectory analysis, to identify latent trajectory groups for each public health measure, inductively regrouping participants with similar patterns of adherence over time^9,10^. A censored normal (CNORM) distribution was used, as it is recommended for modeling “the conditional distribution of psychometric scale data, given group membership”^48^. Contrary to many other longitudinal methods, LCGA does not require for the assumption of homogeneity of variance between time points to be respected^9,10^. Using the Bayesian Information Criterion (BIC), we chose the model that showed the best fit for the data in terms of number of trajectories (see Supplementary Materials, Table 6, 11, 16). We then adjusted the polynomial function for each trajectory (i.e., constant, linear, quadratic, or cubic) and ultimately retained the model that showed the best fit according to the BIC (lowest value). Next, a multinomial logistic regression was carried out for each public health measure to examine how perceived understanding of information and type of used sources were associated with trajectory group membership, while controlling for age, gender, level of education, political identity, region of residence in Canada (provinces) and immigrant status. The first group, that is the group with the lowest adherence trajectory for each public health measure, was used as the reference group to isolate how a low perceived understanding level and the use of informal sources could hinder adherence. These analyses were conducted using the RISK argument in the PROC TRAJ package in SAS^9,10^. All data visualizations were produced using the ggplot2 package in R.

### Use of experimental animals, and human participants

The authors declare that all experimental protocols were approved by *Comité d'éthique de la recherche en éducation et en psychologie* (CEREP) of University of Montreal (Certificat no CEREP-20-038-D).

The authors declare that all methods were carried out in accordance with relevant guidelines and regulations.

The authors declare that informed consent was obtained from all subjects and/or their legal guardians.

## Results

### Modelling latent trajectories

First, we modelled trajectories for the adherence to the social distancing measure. The optimal model, had four trajectories, respectively linear, cubic, cubic and constant (BIC = − 26,344.55; see Fig. 1). More details about model parameters, bivariate statistics for groups of adherence and average group assignment probability can be found in the Supplementary Materials (Table 7, 8, 9). Participants in the first group, used as reference for comparison between trajectories, showed a low and decreasing adherence to social distancing over time (n = 139, 5.3%). Participants in the second and third groups showed a fluctuating level of adherence through time. Participants in the second group started at a medium level of adherence (n = 855, 32.7%), whereas participants of the third group started at a higher level (n = 1018, 39.0%). Finally, participants in the fourth group followed a constant trajectory of high adherence (n = 601, 23.0%).

Second, we modelled trajectories for the adherence to the contacts’ limitation measure, evaluated at all 11 waves of the survey. The optimal model, had four trajectories, all cubic except for the highest adherence one which is constant (BIC = − 28,104.50; see Fig. 2). More details about model parameters, bivariate statistics for groups of adherence and average group assignment probability can be found in the Supplementary Materials (Table 12, 13, 14). Trajectories were very close to those regarding adherence to social distancing. Participants in the first three groups showed a fluctuating level of adherence through time. Participants in the first group started at the lowest level of adherence and showed more variation in adherence through time (n = 156, 6.0%). Participants in the second group started at a higher level of adherence (n = 917, 35.1%), and participants in the third group had a generally higher level of adherence throughout time, though still fluctuating (n = 1027, 39.3%). Finally, participants in the fourth group again followed a constant trajectory of high adherence (n = 513, 19.6%).

Third, we modelled trajectories for the adherence to the mask wearing measure, evaluated at six waves of the survey starting in June 2020. The optimal model, had five trajectories (BIC = − 15,761.18; see Fig. 3). More details about model parameters, bivariate statistics for groups of adherence and average group assignment probability can be found in the Supplementary Materials (Table 17, 18, 19). The first trajectory was linear, the third was constant, and the second, fourth and fifth trajectories were quadratic. Participants in the first group had a low level of adherence that increased slowly to a medium level of adherence (n = 50, 2.5%). Participants in the third group had a constant and medium level of adherence to mask wearing throughout time (n = 137, 6.9%). Finally, participants in the second, fourth and fifth groups all followed trajectories that increased to a very high level of adherence, but with different starting points: low for the second (n = 362, 18.3%), medium for the fourth (n = 779, 39.4%), and high for the fifth (n = 647, 32.8%).

### Examining impact of predictors on group membership

Next, we used multinomial logistic regression for each public health measure to examine how perceived understanding of information and type of used sources were associated with trajectory group membership, while controlling for age, gender, level of education, political identity, region of residence in Canada (provinces) and immigrant status. Table 1 and Fig. 4 show the adjusted odds ratio (AOR) of group membership for the social distancing latent trajectory model. Understanding of information regarding COVID-19 and the use of official sources both show significant associations to adherence trajectories. For understanding, the positive odds ratios indicate that a higher level of understanding is associated with more chances of following higher adherence trajectories. For example, a person with a self-assessed level of understanding of 9 (on the 1–10 scale), compared to a person with an understanding level of 6, would be 2.1 (CI 1.9–2.2) times more likely to be in the “Medium and fluctuating” group, 5.3 (CI 4.9–5.8) times more likely to be in the “High and fluctuating” group and 17.6 (CI 15.4–20.2) more likely to be in the “High and constant” group. Similarly, the use of official sources is positively associated with chances of following increasingly higher adherence trajectories. Specifically, people who use official sources are 2.7 (CI1.8–4.0) times more likely to be in the “Medium and fluctuating” group, 2.9 (CI 1.9–4.6) times more likely to be in the “High and fluctuating” group and 3.4 (CI 2.3–5.1) times more likely to be in the “High and constant” group. For detailed results of associations of predictors and control variables with adherence trajectories, See Supplemental Materials, Table 100.

**Table 1:** Predictors of adherence to social distancing.

Variables	2: Medium and fluctuating (n = 855, 32.7%)	3: High and fluctuating (n = 1018, 39.0%)	4: High and constant (n = 601, 23.0%)
	AOR (95% CI)	AOR (95% CI)	AOR (95% CI)
Level of understanding	1.3 (1.2–1.4)**	1.8 (1.6–1.9)***	2.6 (2.3–3.0)***
Use of official sources	2.7 (1.8–4.0)*	2.9 (1.9–4.6)*	3.4 (2.3–5.1)**

Reference group: *1*—*Low and decreasing* (n = 139, 5.3%).

Controlled for gender, age, education level, region, political identity and immigration status.

*AOR* adjusted odds ratio, *95% CI* 95% confidence interval.

*p < 0.05; **p < 0.01; ***p < 0.001.

Table 2 and Fig. 5 show the adjusted odds ratio of group membership for the contacts’ limitation latent trajectory model. Again, both perceived understanding and type of used sources are significant predictors of adherence. For understanding, the positive odds ratios show that a higher level of understanding is linked to increased chances of following higher adherence trajectories, although there is no distinction between the first and second group. Thus, a person with a self-assessed level of understanding of 9 (on the 1–10 scale), compared to a person with an understanding level of 6, would be 3.0 (CI 2.8–3.3) times more likely to be in the “High and fluctuating” group and 7.8 (CI 6.8–8.8) more likely to be in the “High and constant” group. Similarly, the use of official sources is positively associated with chances of following increasingly higher adherence trajectories. Specifically, people who use official sources are 1.8 (CI 1.4–2.4) times more likely to be in the “Medium and fluctuating” group, 3.3 (CI 2.5–4.2) times more likely to be in the “High and fluctuating” group and 2.9 (CI 2.2–3.9) times more likely to be in the “High and constant” group. For detailed results of associations of predictors and control variables with adherence trajectories, See Supplemental Materials, Table 15.

**Table 2:** Predictors of adherence to contacts limitation.

Variables	2: Medium and fluctuating (n = 917, 35.1%)	3: High and fluctuating (n = 1027, 39.3%)	4: High and constant (n = 513, 19.6%)
	AOR (95% CI)	AOR (95% CI)	AOR (95% CI)
Level of understanding	1.2 (1.1–1.3)	1.4 (1.3–1.6)***	2.0 (1.7–2.3)***
Use of official sources	1.8 (1.4–2.4)*	3.3 (2.5–4.2)***	2.9 (2.2–3.9)***

Reference group: *1—Low and fluctuating* (n = 156, 6.0%).

Controlled for gender, age, education level, region, political identity and immigration status.

*AOR* adjusted odds ratio, *95% CI* 95% confidence interval.

*p < 0.05; **p < 0.01; ***p < 0.001.

Though there were fewer significant relationships, Table 3 and Fig. 6 show the adjusted odds ratio of group membership for the mask wearing latent trajectory model. Only two groups showed significant difference with the reference group: the “Medium and increasing fast”, in relation to the use of official sources, and the “High and constant” group in relation to level of understanding. Indeed, use of official sources only slightly increases chances to be in the “Medium and increasing” group, making 2.9 (CI 1.8–4.5) times more likely. Level of understanding only increased the chances of being in the “High and constant group” slightly, such that a person with a self-assessed level of understanding of 9 (on the 1–10 scale), compared to a person with an understanding level of 6, would be 4.5 (CI 4.0–5.5) times more likely to be in that group. For detailed results of associations of predictors and control variables with adherence trajectories, See Supplemental Materials, Table 20.

**Table 3:** Predictors of adherence to mask wearing.

Variables	2: Low and increasing fast (n = 362, 18.3%)	3: Medium and constant (n = 137, 6.9%)	4: Medium and increasing fast (n = 779, 39.4%)	5: High and constant (n = 647, 32.8%)
	AOR (95% CI)	AOR (95% CI)	AOR (95% CI)	AOR (95% CI)
Level of understanding	1.2 (1.0–1.4)	0.9 (0.7–1.1)	1.3 (1.1–1.5)	1.7 (1.4–2.0)***
Use of official sources	2.4 (1.5–3.9)	1.2 (-0.7–2.0)	2.9 (1.8–4.5)*	2.1 (1.3–3.3)

Reference group: *1—Low and increasing slowly* (n = 50, 2.5%).

Controlled for gender, age, education level, region, political identity and immigration status.

*AOR* adjusted odds ratio, *95% CI* 95% confidence interval.

*p < 0.05; **p < 0.01; ***p < 0.001.

In addition, we noted some interesting results pertaining to the sociodemographic factors used as control variables. Age proved to be a significant predictor for social distancing and contacts’ limitation trajectories, as the mean age increased with each trajectory following level of adherence. In other words, the older people are, the more likely they are to follow public health measures. Political orientation was also a significant factor in predicting lower adherence for all measures, participants in the lowest adherence group being significantly more to the right than participants in higher adherence trajectories. Finally, we observed significant differences depending on Canada's regions for mask wearing, which seems to relate to the beginning of legal enforcement of mask wearing at various moments during the pandemic.

## Discussion

The aim of this research was to examine how individuals’ perceived understanding of information regarding COVID-19 and their use of official sources of information affected their level of adherence to key public health measures in the long term. We used latent trajectories to model adherence levels over the first year of the pandemic and generally found that there was one group of individuals who presented a significantly lower level of adherence to the measures throughout time. This low adherence group included between 2.5 and 6.0% of the population depending on the specific measure considered (5.3% for social distancing, 6.0% for contacts’ limitation and 2.5% for mask wearing). Thus, we start by acknowledging that only a very small proportion of the Canadian population showed extremely low adherence to public health measures. However, this small proportion is enough to hinder the impact of preventive measures^49^, which is why it is so important to understand what influences some individuals to ignore them. Moreover, many studies up to date have focused on sociodemographic variables to detect population differences in adherence levels. Thus, it is important to keep in mind that all results were controlled for variables such as age, gender, education level, political identity, immigrant status and region of Canada.

First, we postulated that individuals who don’t understand information related to COVID-19 and preventive public health measures would be less likely to follow said measures. Our results show that the level of perceived understanding was positively linked to adherence, such that the higher the self-assessed level of understanding was, the higher were the chances of a person following a high adherence trajectory, and vice-versa. Although a high level of understanding does not guarantee a perfect adherence, a lack of comprehension regarding COVID-19 and associated preventive measures hinders adherence. This is not surprising considering that good knowledge and understanding of an epidemic outbreak was proved to be important in encouraging adherence to quarantine compliance in other cases of outbreak of H1N1, SRAS or Ebola in various countries^50^. Although there is little literature on situations comparable to COVID-19, these results are also coherent with what is known to affect patients’ adherence to preventive measures for health concerns in general. Indeed, it is well documented that patients are much less likely to adhere to preventive measures relevant for their specific ailments (whether it is cancer, diabetes, recovering for physical injuries, etc.) when they don’t understand their disease or the role that the preventive measures they are encouraged to implement is supposed to play in the management of their illness^25^. This relation between level of understanding and adherence prompted many medical practitioners, researchers, and associations to advocate for better health literacy in the general population^25–27^. Health literacy, considered as the capacity to understand, access, and use health-related information, is the primary predictor of understanding an illness and the impact of related preventive measures or treatment, regardless of other factors such as education^13,16,29^. Our results simply suggest that this relation holds true in the case of COVID-19, meaning that better health literacy would improve individuals’ level of understanding and thus have a positive influence on adherence to preventive measures. Although this is clearly not something that can be implemented quickly, policies that enhance health-related education in school and in public communications by the government should be a priority not only to improve individuals’ understanding in the case of personal illness, but for matters of public health such as future epidemics. For a more immediate effect, governments and public health agencies should strive to provide clear and coherent communication regarding public health measures if they want to ensure a higher level of understanding and improve adherence.

For the specific results regarding mask wearing, the apparent lack of link between our predictive factors and this important public health measure might lie in methodological and circumstantial limitations of measures. For example, the “Medium and constant” group is composed of participants that are very similar to the lowest adherence group participants in terms of covariates and seems to represent people with a certain reluctance towards public health measures, which might explain the absence of significant differences. We must also acknowledge that other factors that we could not observe and measure probably had a significant influence on adherence. For instance, mask wearing was much more visually noticeable, which may have created a peer pressure effect, incentivizing people to adhere to this measure regardless of their perceived understanding level. Moreover, legal and practical constraints limiting access to certain areas or activities when public health measures weren’t followed (again mostly for mask wearing) definitely had an effect on adherence, independently of the issue of misinformation. Finally, the measure of adherence to mask wearing showed more variation over time than the other two sanitary measures (see Supplemental Materials, Table 3). This measurement issue might also explain the lack of significant statistical relationships between our predictors and mask wearing trajectories.

Furthermore, we believed that use of informal sources of information would be associated with lower adherence. As the results showed, we did find some significant link between the use of informal sources and low adherence to social distancing and contacts’ limitation, but not as much for mask wearing. This is coherent with the results of many other studies that showed reliance on sources such as the government or health agency encouraged adherence while the use of social medias could decrease adherence^14,21,24^. Furthermore, for all three measures, the effect of informal sources on low adherence was much less important than that of the perceived understanding level, being only significant in distinguishing the lowest from the highest adherence trajectories, with odds ratio of ~ 2.

It is also interesting to note that the perceived level of understanding remained an important predictor of adherence regardless of the type of used sources or education level, two factors that are much more highlighted in public and political discourse. This goes to show that while the use of informal sources can be problematic, its effect is usually not as important as the perceived level of understanding. While some research demonstrated that individuals with higher education levels showed better knowledge and understanding of various issues^13^, in our study, education was shown to be less relevant to promoting adherence than knowledge and understanding level. This could potentially be explained by the interaction between education and choices of sources, affecting knowledge together^13,14,18^, an interaction that could be explored in further studies.

Finally, we also include some control variables that despite not being the main subject of this article, offer interesting insight into other factors that relate to adherence. These sociodemographic variables showed no collinearity and were only weakly correlated. Confirming what had been found by many other studies, we noted that being younger and being more to the right of the political spectrum increased risk of following a low adherence trajectory^51–56^. Although the relationship between partisanship and COVID-19 related behaviours is a very interesting topic, partisanship was not one of our main variables of interest in this article. Moreover, partisanship in Canada does not play out the same way as in the United States, because COVID-19 was simply less politicized, though towards the end of the pandemic people on the right crystallized their attitudes against the adherence of health measures^57^. The resistance toward COVID-19 measures on the side of people to the right of the political spectrum in Canada can be explained by at least three phenomena; first that a Centre-left party is in government at the time of COVID-19; second that anti-COVID-19 sentiment was very prevalent in the United States on the political right; thirdly social dominance attitudes are more predictive of non-adherence^58^. We also found significant differences in adherence to mask wearing depending on the Canadian region, which is coherent with the fact that mask wearing was imposed at different moments and at different speeds throughout the country^59^.

This study has significant strengths, most importantly its longitudinal design. We used 11 time points to construct latent trajectories, which gives an accurate and detailed portrait of the evolution of adherence levels during the first year of the pandemic in Canada. This longitudinal design also allowed us to examine the influence of initial perceived level of understanding and type of used sources at the beginning of the pandemic over the long-term. The use of six control variables also allowed us to assess the impact of misinformation of adherence beyond sociodemographic variations in the population.

This study also has limitations. First and foremost, adherence and understanding were both measured based on participants’ perception of their own adherence and understanding. Although results are coherent with previous studies, external measures of adherence would have been a more accurate reflection of people’s behaviour during the pandemic. Similarly, the variable for level of understanding is based on self-assessment of understanding by respondents. An independent measure of understanding through a questionnaire assessing respondent’s knowledge of COVID-19 related fact might have given a more objective measure of understanding. Using variables constructed from participants’ own perception of their behaviour creates a limitation, though the results are still very interesting and relevant when interpreted by acknowledging the self-reported nature of the variables. Moreover, as the measures for concepts related to COVID-19 were developed quickly at the beginning of the pandemic, each scientific team usually developed their own measures, questions, or items. Though all these measures are usually similar and valid, the lack of standardized ways to assess these concepts is an important limitation to the development of consensus in the literature. Finally, the small size of the low-adherence groups used as reference could limit replicability. Results must be interpreted as representative of a general trend.

In this study, we found that over the long-term, there is usually a group of people representing around 5% of the population that displays significantly lower adherence to public health measures. This low adherence group distinguishes itself by its lower level of understanding and to a lesser extent its use of informal sources. Overall, we showed that perceived understanding of information was a key predictor of adherence to all COVID-19 health measures, regardless of the type of sources used by participants. Although the COVID-19 pandemic is coming to its end, similar global health crises may still arise in the future. This study highlights the importance of clear and coherent communication on the part of governments to allow a good level of understanding of the population when faced with new and sometimes complex guidelines. In the near future, it would be interesting to explore the temporal dynamics that exist between predictive factors and public health measures, as some factors may change over time and their effect on adherence may also vary. Understanding the effect of this evolution on adherence would inform public policy that could be generalized to other health crises.

### Supplementary Information

Supplementary Information.

## Data Availability

The data that support the findings of this study are available from the corresponding author upon request.
