# Supplementary material for: How level of understanding and type of used sources relate to adherence to COVID-19 public health measures in Canada
Source: Sci Rep. 2023 Aug 11;13:13065. doi: 10.1038/s41598-023-38824-0 (PMC10421852; doi:10.1038/s41598-023-38824-0)
Supplement: Supplementary file 1 — Supplementary Information. [file 41598_2023_38824_MOESM1_ESM.docx]

Supplementary Materials

for

**How level of understanding and type of sources used relate to adherence to COVID-19 public health measures in Canada**

**Table of content**

**Supplementary 1: Study Materials** 2

***Supplementary 1a. COVID-19: the end of the world as we know it?*** 2

Table 1. Descriptive statistics of the survey 2

***Supplementary 1b. Variables*** 4

*Adherence to public health measures* 4

Table 2. Detailed description of the questions used as dependant variables 4

Figure 1. Correlations between social distancing items at each time point 5

Figure 2. Correlations between contacts’ limitation items at each time point 6

Figure 3. Correlations between mask wearing items at each time point 7

Table 3. Correlations and Cronbach’s alpha for all three public health measures items at each time point 8

*Type of used sources* 9

Figure 4. Results from the iCare Study on the importance of sources of information 9

Table 4. Percentages of top 3 chosen sources of COVID information among 10 10

Figure 5. Correlations between top 3 chosen sources of COVID information among 10 11

*Control variables* 12

Table 5. Descriptive statistics for control variables 12

Figure 6. Correlations between independent variables 13

**Supplementary 2: Results** 14

***Supplementary 2a. Social distancing trajectories*** 14

Table 6. Bayesian information criterion for selection of a trajectory model 14

Table 7. Bivariate statistics for groups of adherence to social distancing 15

Table 8. Detailed results for trajectories of adherence to social distancing (model parameters) 16

Table 9. Average group assignment probability to social distancing group, conditional on assignment by maximum probability 16

Table 10. Associations with predictors and control variables 17

***Supplementary 2b. Contacts’ limitation trajectories*** 19

Table 11. Bayesian information criterion for selection of a trajectory model 19

Table 12. Bivariate statistics for adherence trajectories to contacts limitation 20

Table 13. Detailed results for trajectories of adherence to contacts’ limitation (model parameters) 21

Table 14. Average group assignment probability to contacts’ limitation group, conditional on assignment by maximum probability 21

Table 15. Associations with predictors and control variables 22

***Supplementary 2c. Mask wearing trajectories*** 24

Table 16. Bayesian information criterion for selection of a trajectory model 24

Table 17. Bivariate statistics for adherence trajectories to mask wearing 25

Table 18. Detailed results for trajectories of adherence to social distancing (model parameters) 26

Table 19. Average group assignment probability to mask wearing group, conditional on assignment by maximum probability 26

Table 20. Associations with predictors and control variables 27

**References** 30

# Supplementary 1. Study Materials

This project is part of a longitudinal study on the psychological impacts of COVID-19 on the Canadian population. This project, entitled "COVID-19 Canada: the end of the world as we know it?", was carried out with the collaboration of several researchers from different disciplines. The objective of this project is to understand the impacts on the Canadian population of a dramatic social change such as the COVID-19 pandemic through three themes: adherence to health measures, social cohesion, and well-being. The participants were questioned on more than a hundred variables covering concepts as diverse as emotions, behaviors, attitudes, and cognitions related to the COVID-19 pandemic.

## Supplementary 1a. COVID-19: the end of the world as we know it?

### Table 1. Descriptive statistics of the survey

| Time point | | *Survey dates* | *Sample Size (N)* | *Intervals between time points* | | *Number of weeks elapsed since launch of survey* |
| --- | --- | --- | --- | --- | --- | --- |
| *1* | April 6^th^ – May 6^th^ 2020 | | 3617 | 2 weeks | 0 | |
| *2* | April 21^st^ – May 13^th^ 2020 | | 2282 | 2 weeks | 2 | |
| *3* | May 4^th^ – May 25^th^ 2020 | | 2369 | 2 weeks | 4 | |
| *4* | May 18^th^ – June 10^th^ 2020 | | 2296 | 2 weeks | 6 | |
| *5* | June 1^st^ – June 23^rd^ 2020 | | 2154 | 2 weeks | 8 | |
| *6* | June 15^th^ – July 13^th^ 2020 | | 2116 | 2 weeks | 10 | |
| *7* | July 13^th^ – August 8^th^ 2020 | | 2072 | 4 weeks | 14 | |
| *8* | August 17^th^ – September 13^th^2020 | | 1871 | 5 weeks | 19 | |
| *9* | September 21^st^ – October 19^th^ 2020 | | 1821 | 5 weeks | 24 | |
| *10* | November 26^th^ –December 29^th^ 2020 | | 1883 | 9 weeks | 33 | |
| *11* | April 13^th^ – May 31^st^ 2021 | | 2002 | 20 weeks | 53 | |
| *12* | Mars 2^nd^ – April 19^th^ 2022 | | 1672 | 46 weeks | 99 | |

Table 1 illustrates various elements of the survey: the number of time points, the timeline of the survey, and the sample size. Most importantly, this table indicates the coding scheme for this study, in which we only refer to the number of weeks elapsed since the launch of the survey as time point (from 0 to 53).

## Supplementary 1b. Variables

### Adherence to public health measure

#### Table 2. Detailed description of the questions used as dependant variables

| *Questions from the survey* | *Scale* | *Dates of entry in the survey* |
| --- | --- | --- |
| *Currently, how often do you do the following?* |  |  |
| Maintain a distance of at least two meters (about two arm's lengths) from others when I am not at home | 1-10 (Never to Always) | April 6^th^ – May 6^th^ 2020 |
| Stay home as much as I can | 1-10 (Never to Always) | April 6^th^ – May 6^th^ 2020 |
| Wear a mask in public | 1-10 (Never to Always) | June 1^st^ – June 23^rd^ 2020 |

Participants were questioned on their level of adherence to public health measures by reporting how often they would follow the public health recommendations. Table 2 shows that mask wearing was introduced later in the survey, which explains the time gap with other measures.

#### Figure 1. Correlations between social distancing items at each time point


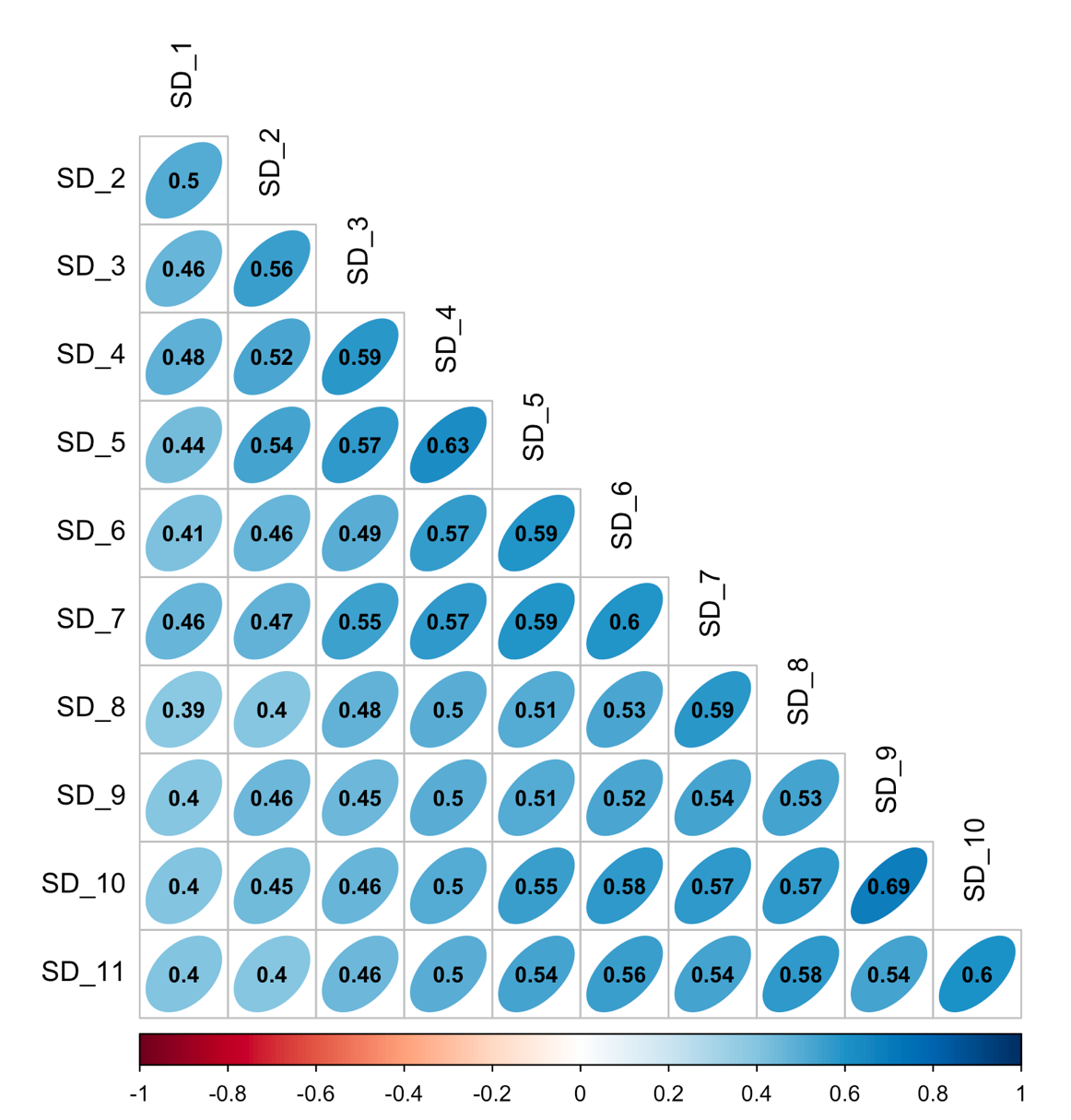


Figure 1 illustrates the Pearson correlations between social distancing variables at all 11 time points. Associations between the same variable at different time measures were evaluated to verify the validity of the question from the survey. Indeed, this allowed us to verify whether the answers from one time point to another were similar in pattern. In the case of social distancing, we can confirm that all variables are strongly associated with one another as correlations range between 0.39 and 0.69.

N.B. All numbers displayed with only one-digit have a zero as the following digit and were automatically rounded by the software.

#### Figure 2. Correlations between contacts limitation items at each time point


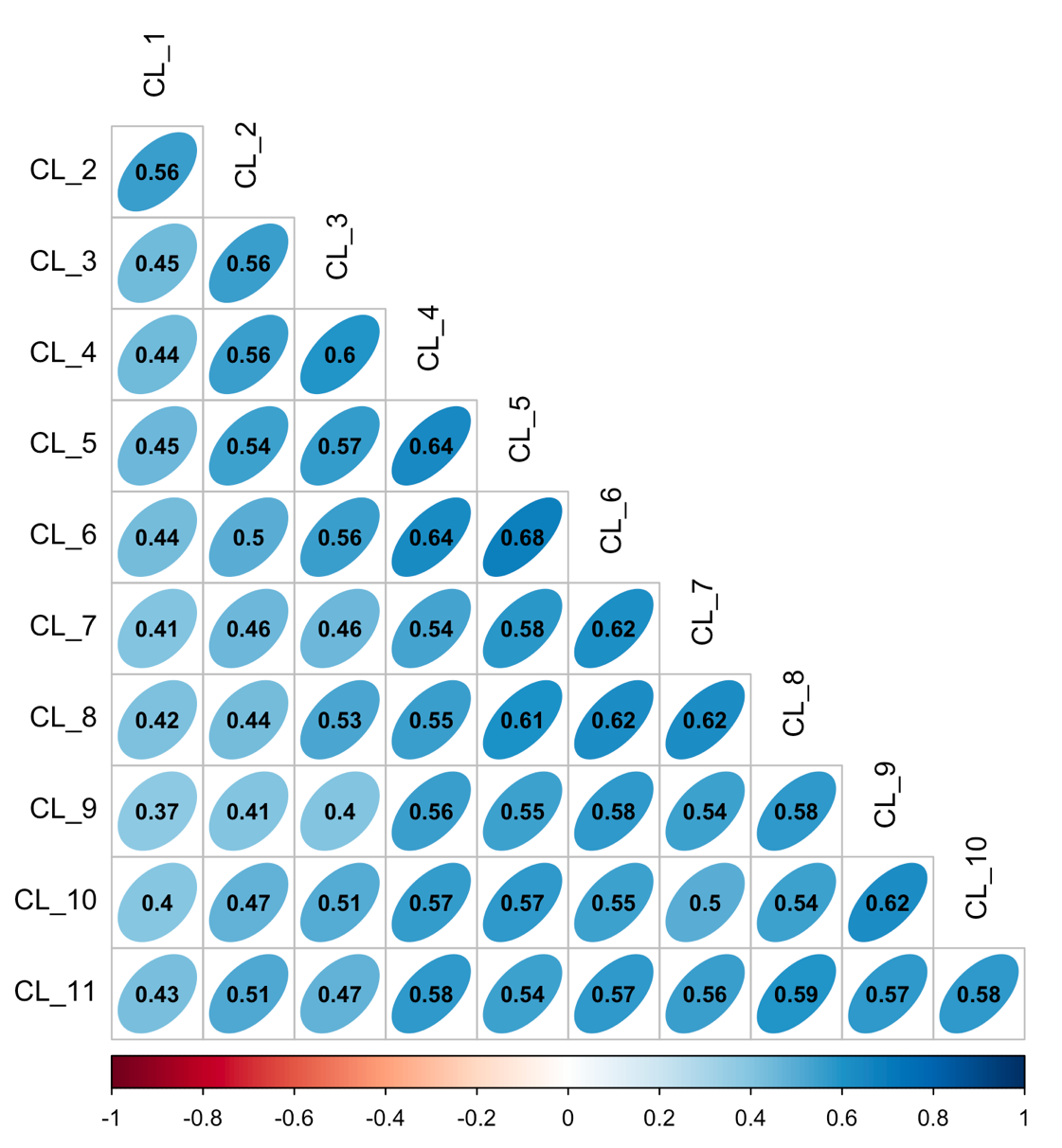


Figure 2 illustrates the Pearson correlations between contacts limitation variables at all 11 time points. The correlations vary between 0.37 and 0.68, which indicates the presence of strong associations between variables of contacts limitations.

N.B. All numbers displayed with only one-digit have a zero as the following digit and were automatically rounded by the software.

#### Figure 3. Correlations between mask wearing items at each time point


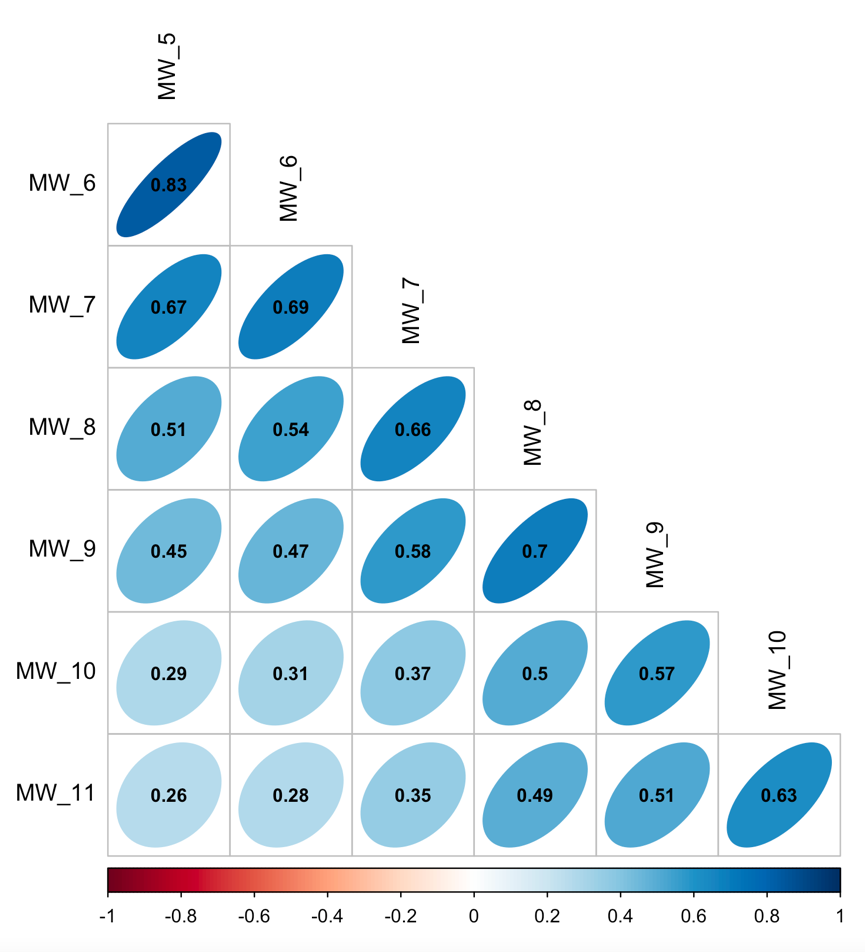


Figure 3 illustrates the Pearson correlations between mask wearing variables at all 6 time points. The correlations vary between 0.26 and 0.83, which indicates the presence of strong associations between variables of mask wearing.

N.B. All numbers displayed with only one-digit have a zero as the following digit and were automatically rounded by the software.

Table 3. Correlations and Cronbach’s alpha for public health measures at each time point

| Time point | Correlation  SD & CL | Correlation  SD & MW | Correlation  CL & MW | Cronbach’s Alpha (CI) |
| --- | --- | --- | --- | --- |
| 1 | .44 | N/A | N/A | .62  (.56–.66) |
| 2 | .44 | N/A | N/A | .62  (.55–.67) |
| 3 | .46 | N/A | N/A | .63  (.58–.68) |
| 4 | .49 | N/A | N/A | .65  (.60–.70) |
| 5 | .49 | .36 | .37 | .61  (.58–.64) |
| 6 | .50 | .38 | .39 | .65  (.62–.68) |
| 7 | .53 | .43 | .33 | .57  (.45–.66) |
| 8 | .49 | .50 | .38 | .66  (.52–.77) |
| 9 | .52 | .50 | .47 | .73  (.58–.83) |
| 10 | .63 | .58 | .47 | .84  (.72–.93) |
| 11 | .63 | .60 | .44 | .80  (.65–.90) |

SD: social distancing

CL: contacts limitation

MW: mask wearing

CI: Confidence Interval

This table illustrates the Pearson correlations between the items for each public health measure at each time point (mask wearing was included only starting at the fifth time point) and the Cronbach’s Alpha between those items. The correlations between different health measures items vary between 0.33 and 0.63, which indicates the presence of medium strong associations between public health measures. Cronbach’s Alpha varies from 0.57 to 0.84, indicating at most time point that the public health measures items could be used to construct one variable, but are still separate, distinctive items.

### Type of used sources

This question was derived from a larger study on COVID-19, the iCare Study (Bacon et al., 2021). In this study, participants were asked: “Among the following sources of information about COVID-19, please rank all the sources that you use, from most to least important.” In the longitudinal survey for the project “COVID-19 Canada: the end of the world as we know it”, we used the same source options, but participants were only asked to rank up to three sources that they “trusted the most to get information regarding COVID-19”. The underlying reasoning put forward by the iCare study (conducted in 14 countries by a team composed of over 100 scientists) is that people will use primarily sources they believe to be trustworthy. Although convenience or easy access to the source of information may also play a role, the results seem to support that people choose sources first and foremost according to their perceived trustworthiness, as more “accessible” or “convenient” sources were ranked low (family and friends, workplace, community, internet), whereas less convenient but potentially more trustworthy sources were ranked higher (health authorities, news, WHO, scientific literature). However, trustworthiness of source as perceived by participant is highly subjective. Thus, we divided sources into official and informal sources by using both our own observation in our survey’s answer and the results of the iCare study.

#### Figure 4. Results from the iCare Study on the importance of sources of information


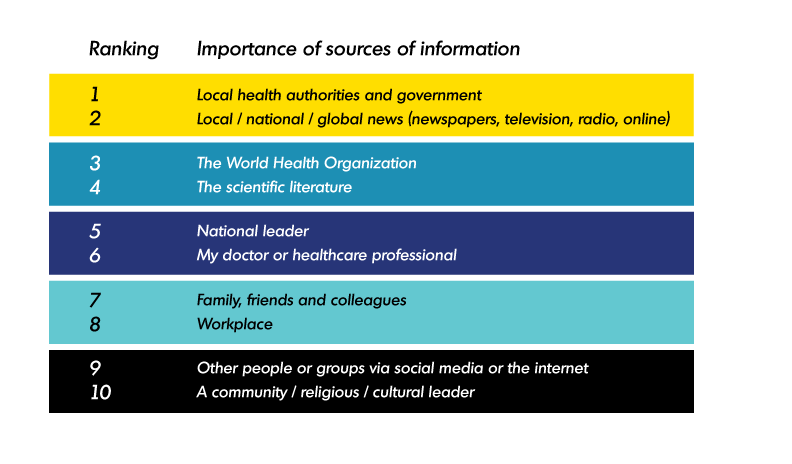


Source : *iCARE Study – Results*. (2021). MBMC. [https://mbmc-cmcm.ca/covid19/research/stats/other/](https://mbmc-cmcm.ca/covid19/research/stats/)

#### Table 4. Percentages of top 3 chosen sources of COVID information among 10

| *Sources* | *1 (%)* | *2 (%)* | *3 (%)* | *In the top 3* | |
| --- | --- | --- | --- | --- | --- |
|  |  |  |  | *Yes (%)* | *No (%)* |
| *Family/friends* | 2 | 3 | 5 | 10 | 90 |
| *Workplace* | 2 | 2 | 3 | 7 | 93 |
| *News* | 21 | 19 | 20 | 60 | 40 |
| *Doctor* | 8 | 11 | 12 | 31 | 69 |
| *Health authorities* | 35 | 30 | 15 | 80 | 20 |
| *Community* | 0,8 | 0,8 | 1,3 | 3 | 97 |
| *WHO* | 18 | 18 | 17 | 52 | 48 |
| *Scientific literature* | 11 | 11 | 12 | 34 | 66 |
| *Others/internet* | 1,5 | 1,5 | 3 | 6 | 94 |

In our preliminary analysis, we found that participants relied mostly on health authorities, news outlets and the WHO to obtain information on the COVID-19 pandemic. Table 4 presents the percentages of sources selected in the top 3, with 80% of participants reporting that health authorities are one the most used sources of information. These results reflect those obtained in the iCare Study, for which the question was originally designed.

#### Figure 5. Correlations between top 3 chosen sources of COVID information among 10


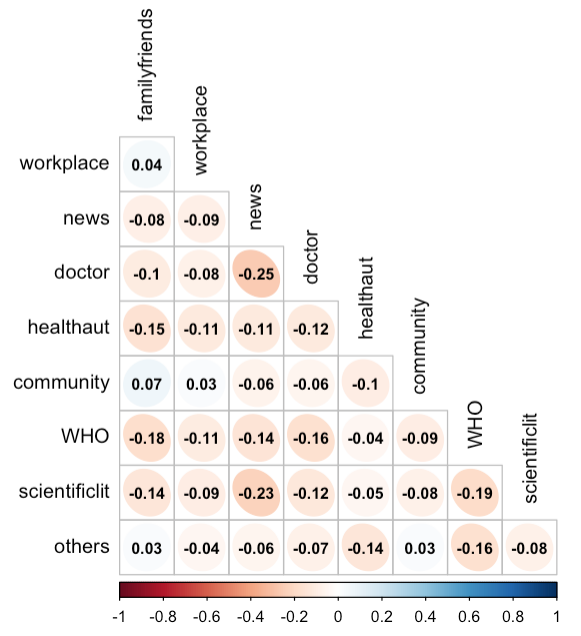


Following the identification of most used sources by participants with the question “Which of the following sources do you trust the most when you want to be informed about COVID-19? Select up to three answers starting with the most trusted source.”, we proceeded to evaluate the correlations between the selected sources. Figure 5 illustrates the degree of correlations between the various sources of information. Unsurprisingly, most sources are negatively correlated with the others, as participants were restrained to choose and rank a maximum of three sources, meaning that each selected source was conjointly selected with only two other sources for each participant. The positive correlations suggest that the two sources were more often selected conjointly by participants as part of their most trusted sources of information, though the positive correlation we observe are very weak. Following this reasoning, we can see that the following pairs of sources were slightly more often selected together: family and friends and workplace; family and friends and community; family and friends and others; workplace and community; community and others. As a reminder, we categorized healthcare authorities, news organisations, the WHO and doctors as official sources. The remaining options, like family/friends, the workplace, the community, and others/Internet, were identified as informal sources. From these preliminary results, we can see that people using informal sources often used them conjointly, whereas people using official sources seldom used them conjointly with an informal source, as shown by negative correlations.

### Control Variables

#### Table 5. Descriptive statistics for control variables

|  | *Sub-sample used in this study (N=2659)* | | *Full original sample (N=3617)* |
| --- | --- | --- | --- |
| *Variables* | *Mean (SD)* | *Weighted mean (SD)* | *Mean*  *(SD)* |
| *Age* | 49.8 (16.6) | 45.4 (15.6) | 48.0 (16.9) |
| *Political identity*  *(1: left, 10: right)* | 5.2 (1.9) | 5.3 (1.9) | 5.2 (1.9) |
|  | *% (n)* | *Weighted %* | *% (n)* |
| *Gender* |  |  |  |
| *Male* | 50.1 (1316) | 50.2 | 50.3 (1778) |
| *Female* | 49.9 (1313) | 49.2 | 49.7 (1759) |
| *Education level* |  |  |  |
| *No diploma* | 2.1 (55) | 2.7 | 2.3 (87) |
| *Secondary school diploma* | 17.3 (454) | 16.0 | 17.1 (606) |
| *College, CEGEP or other non-university diploma* | 28.8 (756) | 29.5 | 30.2 (1068) |
| *Bachelor's degree* | 32.5 (854) | 32.5 | 31.3 (1106) |
| *Master's degree* | 12.4 (325) | 11.7 | 12.1 (429) |
| *Earned doctorate* | 7.0 (185) | 6.7 | 6.8 (241) |
| *Canadian Regions* |  |  |  |
| *Ontario* | 40.5 (1058) | 37.9 | 39.2 (1378) |
| *Atlantic* | 6.8 (177) | 6.2 | 6.8 (239) |
| *Quebec* | 20.3 (532) | 23.6 | 21.0 (739) |
| *Prairies* | 18.0 (474) | 18.2 | 18.1 (636) |
| *BC* | 14.3 (374) | 13.1 | 14.8 (521) |
| *Born in Canada* |  |  |  |
| *Yes* | 79.5 (2077) | 76.9 | 80.0 (2813) |
| *No* | 20.5 (526) | 21.8 | 20.0 (703) |

SD: Standard Deviation

#### Figure 6. Correlations between independent variables


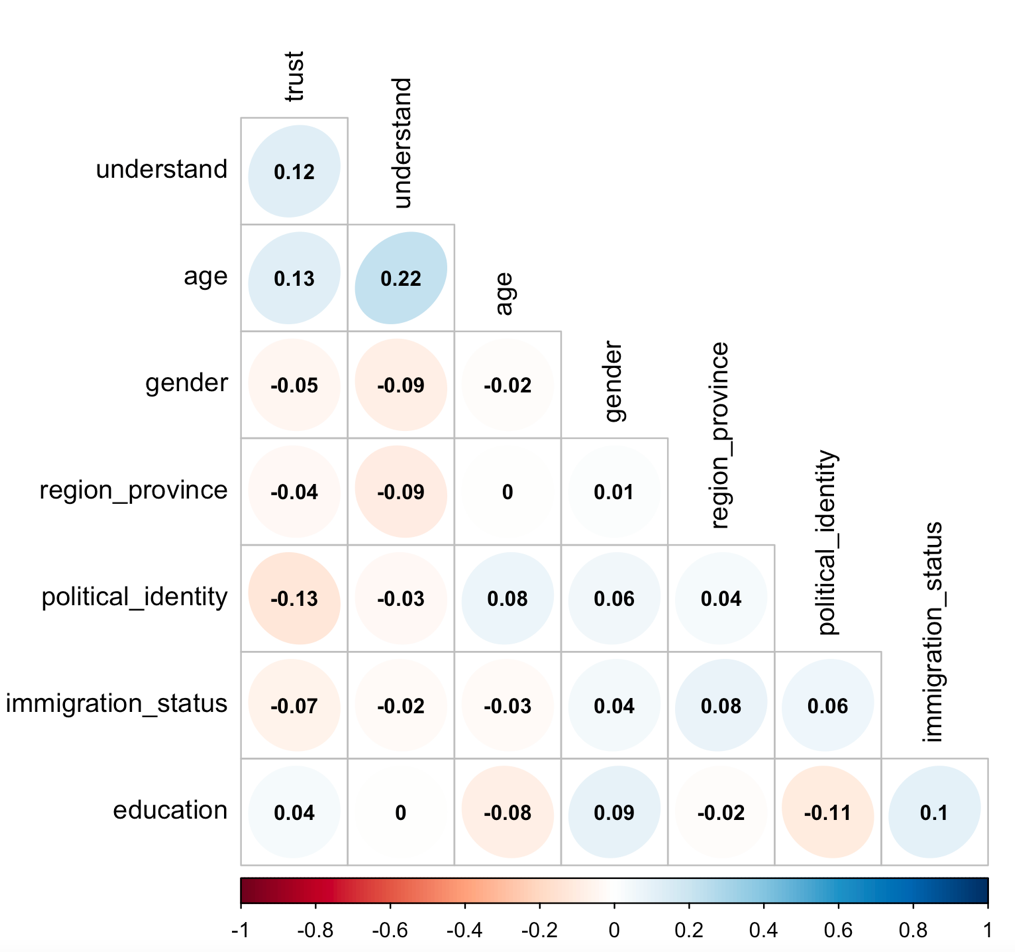


Figure 6 displays the various degrees of associations between independent variables, including both the two main predictors and all control variables. The correlations range from 0 to |0.13|, which reveals that independent variables are not strongly correlated. The only correlation that is slightly higher is between age and perceived understanding (0.22) which may explain why age was often a significant predictor of adherence.

# Supplementary 2. Results

### Supplementary 2A. Social distancing trajectories

#### Table 6. Bayesian information criterion for selection of a trajectory model

| *Model* | *K* | *Order* | *BIC* |
| --- | --- | --- | --- |
| *1* | 1 | 2 | -30 582.30* |
| *2* | 2 | 2,2 | -27 467.71* |
| *3* | 3 | 2,2,2 | -26 595.36* |
| *4* | 4 | 2,2,2,2 | -26 443.93 |
| *5* | 5 | 2,2,2,2,2 | -26 356.12 |
| *6* | 6 | 2,2,2,2,2,2 | -26 375.61 |
| *7* | 4 | 1,2,2,2 | -26 448.29 |
| *8* | 4 | 1,2,2,1 | -26 449.99* |
| *9* | 4 | 1,2,2,0 | -26 449.64* |
| *10* | 4 | 1,3,2,0 | -26 379.29* |
| ***11*** | **4** | **1,3,3,0** | **-26 344.55*** |

Table 6 shows the various attempts at obtaining the best model of social distancing trajectories. Only models with an * were retained for comparison, as the parameters’ estimates were significant (*p* < 0.05). Models in which the smallest group included less than 2% of the sample (above four groups) were not considered. The models were compared according to the Bayesian information criterion (BIC): indeed, the solution with the lowest absolute value of BIC was selected. In this case, we selected the model with 4 trajectories (1,3,3,0).

Table 7. Bivariate statistics for groups of adherence to social distancing

| *Group* | *1 – Low & decreasing  (n = 139, 5.3%)* | | *2: Medium & fluctuating  (n=855, 32.7%)* | *3: High & fluctuating  (n=1018, 39.0%)* | *4: High & constant (n=601, 23.0%)* | *p-value for ANOVA’s F statistic* |
| --- | --- | --- | --- | --- | --- | --- |
| *Variables* | | *Mean (SD)* | | | |  |
| *Understanding  (1: low, 10: high)* | 6.9 (2.1) | | 7.8 (1.5) | 8.6 (1.3) | 9.1 (1.3) | 0.000 *** |
| *Age* | 36.7 (12.0) | | 41.7 (13.8) | 51.1 (15.1) | 51.2 (15.6) | 0.000 *** |
| *Political identity*  *(1: left, 10: right)* | 6.0 (1.9) | | 5.1 (1.8) | 5.2 (1.9) | 5.2 (1.9) | 0.307 |
|  | | *% (n)* | | | | *p-value for χ^2^ joint test of significance* |
| *Use of official sources (Yes)* | 44.6 (62) | | 75.1 (642) | 82.0 (835) | 82.4 (495) | 0.000 *** |
| *Gender (Male)* | 61.2 (85) | | 57.1 (488) | 48.1 (490) | 40.4 (243) | 0.000 *** |
| *Education level* |  | |  |  |  | 0.000 *** |
| *No diploma* | 2.9 (4) | | 1.1 (9) | 2.7 (27) | 2.5 (15) |  |
| *Secondary school diploma* | 21.6 (30) | | 14.2 (121) | 17.4 (177) | 20.6 (124) |  |
| *College, CEGEP or other non-university diploma* | 29.5 (41) | | 30.4 (260) | 25.4 (259) | 32.3 (194) |  |
| *Bachelor's degree* | 28.8 (40) | | 37.3 (319) | 31.4 (320) | 28.1 (169) |  |
| *Master's degree* | 10.8 (15) | | 12.3 (105) | 14.0 (143) | 9.8 (59) |  |
| *Earned doctorate* | 6.5 (9) | | 4.8 (41) | 9.0 (92) | 6.7 (40) |  |
| *Canadian Regions* |  | |  |  |  | 0.000 *** |
| *Ontario* | 33.1 (46) | | 35.8 (304) | 41.5 (421) | 46.8 (279) |  |
| *Atlantic* | 4.3 (6) | | 4.1 (35) | 8.2 (83) | 8.6 (51) |  |
| *Quebec* | 18.7 (26) | | 20.5 (174) | 19.9 (202) | 21.6 (129) |  |
| *Prairies* | 28.1 (39) | | 22.0 (187) | 17.5 (177) | 11.4 (68) |  |
| *BC* | 15.8 (22) | | 17.6 (150) | 12.9 (131) | 11.6 (69) |  |
| *Born in Canada (Yes)* | 77.0 (107) | | 82.9 (709) | 78.0 (794) | 77.7 (467) | 0.030 * |

*p< 0.05 **p< 0.01 *** p< 0.001

#### Table 8. Detailed results for trajectories of adherence to social distancing (model parameters)

| *Group* | *Parameter* | *β* | *SE* | *t* | *p-value* |
| --- | --- | --- | --- | --- | --- |
| *1* | Intercept | 5.871 | .379 | 15.489 | .000 |
|  | Linear | -.029 | .011 | -2.603 | .009 |
| *2* | Intercept | 8.759 | .290 | 30.204 | .000 |
|  | Linear | -.177 | .025 | -7.088 | .000 |
|  | Quadratic | .008 | .001 | 6.380 | .000 |
|  | Cubic | -.000 | .000 | -5.618 | .000 |
| *3* | Intercept | 10.826 | .551 | 19.661 | .000 |
|  | Linear | -.226 | .039 | -5.746 | .000 |
|  | Quadratic | .009 | .001 | 5.790 | .000 |
|  | Cubic | -.000 | .000 | -5.354 | .000 |
| *4* | Intercept | 12.463 | .275 | 45.278 | .000 |

*Note.* *β*: Standardized coefficients

SE: Standard Errors

#### Table 9. Average group assignment probability to social distancing group, conditional on assignment by maximum probability

| Group assignment | Probability (%) | | | |
| --- | --- | --- | --- | --- |
|  | 1 | 2 | 3 | 4 |
| 1 | 91.0 | 0.2 | .0 | .0 |
| 2 | 9.0 | 87.2 | 10.2 | .0 |
| 3 | .0 | 10.6 | 84.8 | 8.0 |
| 4 | .0 | .0 | 4.9 | 92.0 |

#### Overall average group assignment probability to assigned group: 88.8%

#### Table 10. Associations with predictors and control variables

| *Variable* | *Group* | *β* | *SE* | *t* | *p-value* |
| --- | --- | --- | --- | --- | --- |
| *Constant* | 2 | -1.041 | 1.059 | -.983 | .325 |
|  | 3 | -3.978 | 1.538 | -2.587 | .010 |
|  | 4 | -7.995 | 1.777 | -4.498 | .000 |
| *Level of understanding* | 2 | .239 | 0.078 | 3.073 | .002 |
|  | 3 | .557 | .082 | 6.807 | .000 |
|  | 4 | .957 | .135 | 7.076 | .000 |
| *Use of official sources* | 2 | .991 | 0.408 | 2.431 | .015 |
|  | 3 | 1.077 | .456 | 2.362 | .018 |
|  | 4 | 1.221 | .406 | 3.008 | .002 |
| *Age* | 2 | .029 | .010 | 2.800 | .005 |
|  | 3 | .067 | .009 | 7.614 | .000 |
|  | 4 | .064 | .010 | 6.373 | .000 |
| *Gender – Male* | 2 | .103 | 0.357 | .288 | .871 |
|  | 3 | -.294 | .300 | -0.980 | .327 |
|  | 4 | -.540 | .317 | -1.704 | .088 |
| *Region of residence*  *– Atlantic* | 2 | .432 | .761 | .568 | .570 |
|  | 3 | .929 | .629 | 1.477 | .140 |
|  | 4 | .663 | .653 | 1.016 | .310 |
| *Region of residence*  *– Quebec* | 2 | -.278 | .416 | -.667 | .504 |
|  | 3 | -.586 | .381 | -1.539 | .124 |
|  | 4 | -.695 | .419 | -1.600 | .097 |
| *Region of residence*  *– Prairies* | 2 | -.344 | .338 | -1.020 | .308 |
|  | 3 | -.821 | .349 | -2.352 | .019 |
|  | 4 | -1.408 | .374 | -3.762 | .000 |
| *Region of residence*  *– British Columbia* | 2 | -.115 | .387 | -.297 | .767 |
|  | 3 | -.761 | .465 | -1.636 | .102 |
|  | 4 | -1.036 | .405 | -2.557 | .097 |
| *Immigrant* | 2 | .099 | .363 | .271 | .786 |
|  | 3 | .453 | .346 | 1.310 | .190 |
|  | 4 | .648 | .336 | 1.932 | .053 |
| *Education level*  *– High school* | 2 | .126 | .870 | .145 | .885 |
|  | 3 | -.698 | .909 | -.767 | .443 |
|  | 4 | -.289 | .949 | -.304 | .761 |
| *Education level*  *– College* | 2 | .452 | .862 | .524 | .600 |
|  | 3 | -.938 | .865 | -1.084 | .278 |
|  | 4 | -.688 | .897 | -.767 | .443 |
| *Education level*  *– Bachelor* | 2 | .898 | .874 | 1.028 | .304 |
|  | 3 | -.296 | .846 | -.350 | .726 |
|  | 4 | -.224 | .888 | -.252 | .801 |
| *Education level*  *– Master* | 2 | .296 | .904 | .327 | .743 |
|  | 3 | -.675 | .958 | -.705 | .481 |
|  | 4 | -.744 | 1.026 | -.725 | .469 |
| *Education level*  *– Doctorate* | 2 | -.171 | 1.103 | -.155 | .877 |
|  | 3 | -.651 | 1.079 | -.603 | .546 |
|  | 4 | -.743 | 1.103 | -.674 | .500 |
| *Political identity* | 2 | -.230 | .077 | -2.988 | .002 |
|  | 3 | -.223 | .096 | -2.310 | .021 |
|  | 4 | -.214 | .095 | -2.256 | .024 |

*Note.* *β*: Standardized coefficients of multinomial logistic regression

SE: Standard Errors

### Supplementary 2b. Contacts’ limitation trajectories

#### Table 11. Bayesian information criterion for selection of a trajectory model

| *Model* | *K* | *Order* | *BIC* |
| --- | --- | --- | --- |
| *1* | 1 | 2 | -32 696.63* |
| *2* | 2 | 2,2 | -29 472.33* |
| *3* | 3 | 2,2,2 | -28 631.99* |
| *4* | 4 | 2,2,2,2 | -28 383.46* |
| *5* | 5 | 2,2,2,2,2 | -28 284.00 |
| *6* | 6 | 2,2,2,2,2,2 | -28 317.39 |
| *7* | 4 | 2,2,2,1 | -28 390.28 |
| *8* | 4 | 2,2,2,0 | -28 386.83* |
| *9* | 4 | 3,2,2,0 | -28 306.14* |
| *10* | 4 | 3,3,2,0 | -28 198.56* |
| ***11*** | **4** | **3,3,3,0** | **-28 104.50*** |

Table 11 shows the various attempts at obtaining the best model of contacts’ limitation trajectories. Only models with an * were retained for comparison, as the parameters’ estimates were significant (*p* < 0.05). Models in which the smallest group included less than 2% of the sample (above four groups) were not considered. The models were compared according to the Bayesian information criterion (BIC): indeed, the solution with the lowest absolute value of BIC was selected. In this case, we selected the model with 4 trajectories (3,3,3,0).

Table 12. Bivariate statistics for adherence trajectories to contacts limitation

| *Group* | *1 – Low & fluctuating (n=156, 6.0%)* | | *2: Medium & fluctuating (n=917, 35.1%)* | *3: High & fluctuating (n=1027, 39.3%)* | *4: High & constant (n=513, 19.6%)* | *p-value for ANOVA’s F statistic* |
| --- | --- | --- | --- | --- | --- | --- |
| *Variables* | | *Mean (SD)* | | | |  |
| *Understanding  (1: low, 10: high)* | 7.4 (2.1) | | 8.0 (1.4) | 8.6 (1.4) | 9.0 (1.3) | 0.000 *** |
| *Age* | 41.6 (13.2) | | 46.0 (14.7) | 48.5 (16.0) | 47.8 (16.1) | 0.000 *** |
| *Political identity*  *(1: left, 10: right)* | 6.0 (2.0) | | 5.3 (1.8) | 5.1 (1.9) | 5.1 (2.0) | 0.000 *** |
|  | | *% (n)* | | | | *p-value for χ^2^ joint test of significance* |
| *Use of official sources (Yes)* | 54.5 (85) | | 73.6 (675) | 83.3 (856) | 81.5 (418) | 0.000 *** |
| *Gender (Male)* | 59.6 (93) | | 60.3 (553) | 46.8 (481) | 34.9 (179) | 0.000 *** |
| *Education level* |  | |  |  |  | 0.020 * |
| *No diploma* | 1.9 (3) | | 2.4 (22) | 2.5 (26) | 0.8 (4) |  |
| *Secondary school diploma* | 21.8 (34) | | 18.8 (172) | 14.7 (151) | 18.5 (95) |  |
| *College, CEGEP or other non-university diploma* | 32.1 (50) | | 25.8 (237) | 31.5 (323) | 28.1 (144) |  |
| *Bachelor's degree* | 25.6 (40) | | 33.3 (305) | 33.7 (346) | 30.6 (157) |  |
| *Master's degree* | 11.5 (18) | | 12.1 (111) | 12.1 (124) | 13.5 (69) |  |
| *Earned doctorate* | 7.1 (11) | | 7.6 (70) | 5.6 (57) | 8.6 (44) |  |
| *Canadian Regions* |  | |  |  |  | 0.000 *** |
| *Ontario* | 33.8 (52) | | 34.7 (316) | 43.5 (445) | 46.3 (237) |  |
| *Atlantic* | 4.5 (7) | | 5.4 (49) | 8.0 (82) | 7.2 (37) |  |
| *Quebec* | 20.8 (32) | | 23.2 (211) | 18.5 (189) | 19.4 (99) |  |
| *Prairies* | 27.9 (43) | | 19.5 (178) | 16.8 (172) | 15.3 (78) |  |
| *BC* | 13.0 (20) | | 17.2 (157) | 13.2 (135) | 11.7 (60) |  |
| *Born in Canada (Yes)* | 76.9 (120) | | 82.4 (756) | 79.7 (819) | 74.5 (382) | 0.004 ** |

*p< 0.05 **p< 0.01 *** p< 0.001

#### Table 13. Detailed results for trajectories of adherence to contacts’ limitation (model parameters)

| *Group* | *Parameter* | *β* | *SE* | *t* | *p-value* |
| --- | --- | --- | --- | --- | --- |
| *1* | Intercept | 7.014 | .491 | 14.278 | .000 |
|  | Linear | -.528 | .066 | -7.967 | .000 |
|  | Quadratic | .023 | .004 | 6.454 | .000 |
|  | Cubic | -.000 | .000 | -5.716 | .000 |
| *2* | Intercept | 8.995 | .228 | 36.266 | .000 |
|  | Linear | -.305 | .028 | -11.053 | .000 |
|  | Quadratic | .012 | .001 | 8.457 | .000 |
|  | Cubic | -.000 | .000 | -6.982 | .000 |
| *3* | Intercept | 11.386 | .268 | 42.533 | .000 |
|  | Linear | -.341 | .023 | -14.984 | .000 |
|  | Quadratic | .014 | .001 | 12.587 | .000 |
|  | Cubic | -.000 | .000 | -10.571 | .000 |
| *4* | Intercept | 13.051 | .199 | 65.486 | .000 |

*Note.* *β*: Standardized coefficients

SE: Standard Errors

#### Table 14. Average group assignment probability to contacts’ limitation group, conditional on assignment by maximum probability

| Group assignment | Probability (%) | | | |
| --- | --- | --- | --- | --- |
|  | 1 | 2 | 3 | 4 |
| 1 | 88.8 | 3.0 | .0 | .0 |
| 2 | 11.2 | 87.8 | 8.5 | .0 |
| 3 | .0 | 9.2 | 86.9 | 9.3 |
| 4 | .0 | .0 | 4.6 | 90.6 |

Overall average group assignment probability to assigned group: 88.5%

#### Table 15. Associations with predictors and control variables

| *Variable* | *Group* | *β* | *SE* | *t* | *p-value* |
| --- | --- | --- | --- | --- | --- |
| *Constant* | 2 | 0.751 | 1.272 | .590 | .555 |
|  | 3 | 0.749 | 1.355 | .553 | .580 |
|  | 4 | -4.827 | 1.838 | -2.627 | .009 |
| *Level of understanding* | 2 | .157 | .095 | 1.646 | .010 |
|  | 3 | .368 | .090 | 4.071 | .000 |
|  | 4 | .683 | .130 | 5.245 | .00 |
| *Use of official sources* | 2 | .589 | .282 | 2.089 | .037 |
|  | 3 | 1.179 | .255 | 4.631 | .000 |
|  | 4 | 1.074 | .287 | 3.738 | .000 |
| *Age* | 2 | .019 | .007 | 2.814 | .005 |
|  | 3 | .025 | .007 | 3.477 | .000 |
|  | 4 | .018 | .008 | 2.230 | .026 |
| *Gender – Male* | 2 | .010 | .271 | .035 | .972 |
|  | 3 | -.405 | .256 | -1.584 | .113 |
|  | 4 | -.815 | .275 | -2.965 | .003 |
| *Region of residence*  *– Atlantic* | 2 | .137 | .641 | .214 | .831 |
|  | 3 | .284 | .612 | .464 | .643 |
|  | 4 | .283 | .630 | .448 | .640 |
| *Region of residence*  *– Quebec* | 2 | -.288 | .344 | -.838 | .402 |
|  | 3 | -.780 | .349 | -2.232 | .026 |
|  | 4 | -.739 | .377 | -1.961 | .050 |
| *Region of residence*  *– Prairies* | 2 | -.384 | .486 | -.791 | .429 |
|  | 3 | -.712 | .399 | -1.785 | .074 |
|  | 4 | -.859 | .421 | -2.042 | .041 |
| *Region of residence*  *– British Columbia* | 2 | .062 | .417 | .148 | .823 |
|  | 3 | -.481 | .410 | -1.173 | .241 |
|  | 4 | -.638 | .427 | -1.493 | .136 |
| *Immigrant* | 2 | -.190 | .323 | -.587 | .558 |
|  | 3 | .042 | .309 | .136 | .892 |
|  | 4 | .612 | .322 | 1.903 | .057 |
| *Education level*  *– High school* | 2 | -.421 | 1.061 | -.396 | .692 |
|  | 3 | -.753 | .998 | -.754 | .451 |
|  | 4 | .601 | 1.318 | .456 | .648 |
| *Education level*  *– College* | 2 | -.612 | 1.192 | -.513 | .608 |
|  | 3 | -.593 | 1.084 | -.547 | .584 |
|  | 4 | .252 | 1.397 | .181 | .857 |
| *Education level*  *– Bachelor* | 2 | .154 | 1.023 | .151 | .880 |
|  | 3 | -.148 | .941 | -.158 | .875 |
|  | 4 | .867 | 1.25 | .696 | .486 |
| *Education level*  *– Master* | 2 | -.391 | 1.176 | -.333 | .739 |
|  | 3 | -.662 | 1.035 | -.640 | .522 |
|  | 4 | .616 | 1.392 | .443 | .658 |
| *Education level*  *– Doctorate* | 2 | -.327 | 1.247 | -.262 | .793 |
|  | 3 | -.899 | 1.034 | -.640 | .522 |
|  | 4 | .624 | 1.487 | .420 | .675 |
| *Political identity* | 2 | -.197 | .081 | -2.432 | .015 |
|  | 3 | -.899 | 1.117 | -0.804 | .421 |
|  | 4 | -.239 | .092 | -2.599 | .009 |

*Note.* *Β*: Standardized coefficients of multinomial logistic regression

SE : Standard Errors

### Supplementary 2c. Mask wearing trajectories

#### Table 16. Bayesian information criterion for selection of a trajectory model

| *Model* | *K* | *Order* | *BIC* |
| --- | --- | --- | --- |
| *1* | 1 | 2 | -18 157.69* |
| *2* | 2 | 2,2 | -16 470.32* |
| *3* | 3 | 2,2,2 | -15 988.87* |
| *4* | 4 | 2,2,2,2 | -15 814.35* |
| *5* | 5 | 2,2,2,2,2 | -15 644.83 |
| *6* | 6 | 2,2,2,2,2,2 | -15 580.94 |
| *7* | 7 | 2,2,2,2,2,2,2 | -15 557.67 |
| *8* | 5 | 1,2,2,2,2 | -15 647.44 |
| *9* | 5 | 1,2,1,2,2 | -15 741.80 |
| ***10*** | **5** | **1,2,0,2,2** | **-15 761.18*** |

Table 16 shows the various attempts at obtaining the best model of mask wearing trajectories. Only models with an * were retained for comparison, as the parameters’ estimates were significant (*p* < 0.05). The models were compared according to the Bayesian information criterion (BIC): indeed, the solution with the lowest absolute value of BIC was selected. In this case, we selected the model with 5 trajectories (1,2,0,2,2).

Table 17. Bivariate statistics for adherence trajectories to mask wearing

| *Group* | *1 – Low & increasing slowly (n = 50, 2.5%)* | | *2: Low & increasing fast (n=362, 18.3%)* | *3: Medium & constant (n=137, 6.9%)* | *4: Medium & increasing fast (n=779, 39.4%)* | *5: High & constant (n=647, 32.8%)* | *p-value for ANOVA’s F statistic* |
| --- | --- | --- | --- | --- | --- | --- | --- |
| *Variables* | | *Mean (SD)* | | | |  |  |
| *Understanding  (1: low, 10: high)* | 7.5 (2.4) | | 8.2 (1.5) | 7.1 (2.0) | 8.5 (1.3) | 8.9 (1.3) | 0.000 *** |
| *Age* | 45.2 (12.5) | | 44.4 (14.6) | 41.6 (12.6) | 47.4 (15.9) | 50.9 (16.2) | 0.000 *** |
| *Political identity*  *(1: left, 10: right)* | 6.7 (2.2) | | 5.4 (1.8) | 5.9 (1.8) | 4.9 (1.8) | 5.2 (2.0) | 0.000 *** |
|  | | *% (n)* | | | |  | *p-value for χ^2^ joint test of significance* |
| *Use of official sources (Yes)* | 50.0 (25) | | 76.0 (275) | 54.7 (75) | 83.8 (653) | 80.7 (522) | 0.000 *** |
| *Gender (Male)* | 52.0 (26) | | 57.2 (207) | 84.7 (116) | 49.9 (389) | 41.3 (267) | 0.000 *** |
| *Education level* |  | |  |  |  |  | 0.000 *** |
| *No diploma* | 4.0 (2) | | 3.0 (11) | 1.5 (2) | 2.1 (16) | 1.2 (8) |  |
| *Secondary school diploma* | 24.0 (12) | | 22.4 (81) | 5.1 (7) | 15.1 (118) | 17.5 (113) |  |
| *College, CEGEP or other non-university diploma* | 38.0 (19) | | 29.8 (108) | 24.8 (34) | 29.4 (229) | 27.2 (176) |  |
| *Bachelor's degree* | 20.0 (10) | | 30.1 (109) | 44.5 (61) | 31.8 (248) | 33.4 (216) |  |
| *Master's degree* | 4.0 (2) | | 8.8 (32) | 14.6 (20) | 14.9 (116) | 12.5 (81) |  |
| *Earned doctorate* | 10.0 (5) | | 5.8 (21) | 9.5 (13) | 6.7 (52) | 8.2 (53) |  |
| *Canadian Regions* |  | |  |  |  |  | 0.000 *** |
| *Ontario* | 20.4 (10) | | 18.3 (65) | 55.5 (76) | 40.5 (314) | 54.1 (349) |  |
| *Atlantic* | 0.0 (0) | | 9.0 (32) | 2.2 (3) | 8.5 (66) | 6.0 (39) |  |
| *Quebec* | 20.4 (10) | | 16.6 (59) | 16.8 (23) | 15.1 (117) | 22.2 (143) |  |
| *Prairies* | 42.9 (21) | | 32.9 (117) | 5.1 (7) | 22.4 (174) | 8.4 (54) |  |
| *BC* | 16.3 (8) | | 23.3 (83) | 20.4 (28) | 13.5 (105) | 9.3 (60) |  |
| *Born in Canada (Yes)* | 70.0 (35) | | 89.2 (323) | 69.3 (95) | 81.6 (636) | 75.0 (485) | 0.000 *** |

*p< 0.05 **p< 0.01 *** p< 0.001

#### Table 18. Detailed results for trajectories of adherence to social distancing (model parameters)

| *Group* | *Parameter* | *β* | *SE* | *t* | *p-value* |
| --- | --- | --- | --- | --- | --- |
| *1* | Intercept | -2.112 | .813 | -2.596 | .009 |
|  | Linear | 0.162 | .024 | 6.646 | .000 |
| *2* | Intercept | -5.541 | .782 | -7.084 | .000 |
|  | Linear | .835 | .058 | 14.494 | .000 |
|  | Quadratic | -.010 | .000 | -12.844 | .000 |
| *3* | Intercept | 7.379 | .347 | 21.268 | .000 |
| *4* | Intercept | 2.410 | .755 | 3.189 | .001 |
|  | Linear | .503 | .054 | 9.383 | .000 |
|  | Quadratic | -.006 | .000 | -8.834 | .000 |
| *5* | Intercept | 7.231 | .594 | 12.166 | .000 |
|  | Linear | .505 | .064 | 7.854 | .000 |
|  | Quadratic | -.007 | .001 | -6.952 | .000 |

*Note.* *Β*: Standardized coefficients

SE : Standard Errors

#### Table 19. Average group assignment probability to mask wearing group, conditional on assignment by maximum probability

| Group assignment | Probability (%) | | | | |
| --- | --- | --- | --- | --- | --- |
|  | 1 | 2 | 3 | 4 | 5 |
| 1 | 91.9 | 2.2 | .1 | .0 | .0 |
| 2 | 7.3 | 86.7 | 4.0 | 3.8 | .0 |
| 3 | .8 | 2.1 | 81.4 | 4.9 | .5 |
| 4 | .0 | 9.0 | 14.1 | 85.1 | 8.2 |
| 5 | .0 | .0 | .3 | 6.3 | 91.2 |

Overall average group assignment probability to assigned group: 87%

#### Table 20. Associations with predictors and control variables

| *Variable* | *Group* | *β* | *SE* | *t* | *p-value* |
| --- | --- | --- | --- | --- | --- |
| *Constant* | 2 | 1.702 | 1.593 | 1.075 | .282 |
|  | 3 | 2.276 | 2.456 | 1.122 | .262 |
|  | 4 | 1.825 | 1.548 | 1.179 | .262 |
|  | 5 | 1.656 | 1.653 | 1.002 | .317 |
| *Level of understanding* | 2 | .193 | 1.153 | 1.266 | .208 |
|  | 3 | -.094 | 1.164 | .-.575 | .566 |
|  | 4 | .280 | .151 | 1.853 | .064 |
|  | 5 | .515 | .157 | 3.284 | .001 |
| *Use of official sources* | 2 | .869 | .503 | 1.729 | .084 |
|  | 3 | .142 | .545 | .261 | .794 |
|  | 4 | .056 | .456 | 2.318 | .021 |
|  | 5 | .729 | .466 | 1.563 | .118 |
| *Age* | 2 | .000 | .013 | .021 | .983 |
|  | 3 | -.001 | .018 | -.086 | .931 |
|  | 4 | .015 | .013 | 1.184 | .236 |
|  | 5 | .030 | .012 | 2.405 | .016 |
| *Gender – Male* | 2 | .651 | .673 | .967 | .334 |
|  | 3 | 1.762 | .663 | 2.656 | .008 |
|  | 4 | .270 | .575 | .470 | .638 |
|  | 5 | -.047 | .603 | -.078 | .937 |
| *Region of residence*  *– Atlantic* | 2 | 13.671 | 2.441 | 5.601 | .000 |
|  | 3 | 11.989 | 2.571 | 4.664 | .000 |
|  | 4 | 12.823 | 2.377 | 5.394 | .000 |
|  | 5 | 12.129 | 2.372 | 5.113 | .000 |
| *Region of residence*  *– Quebec* | 2 | -.406 | .622 | -.653 | .514 |
|  | 3 | -1.234 | .644 | -1.917 | .055 |
|  | 4 | -1.183 | .573 | -2.065 | .039 |
|  | 5 | -1.110 | .572 | -1.940 | .052 |
| *Region of residence*  *– Prairies* | 2 | -.219 | .591 | -.372 | .710 |
|  | 3 | -3.638 | 1.253 | -2.902 | .004 |
|  | 4 | -1.478 | .561 | -2.634 | .008 |
|  | 5 | -2.622 | .567 | -4.625 | .000 |
| *Region of residence*  *– British Columbia* | 2 | .411 | .814 | .505 | .614 |
|  | 3 | -.735 | .840 | -.875 | .382 |
|  | 4 | -.943 | .797 | -1.183 | .237 |
|  | 5 | -1.699 | .771 | -2.203 | .028 |
| *Immigrant* | 2 | -1.220 | .579 | -2.107 | .035 |
|  | 3 | -.339 | .606 | -.560 | .576 |
|  | 4 | -.459 | .548 | -.837 | .402 |
|  | 5 | -.005 | .524 | -.009 | .923 |
| *Education level*  *– High school* | 2 | -.191 | 1.227 | -.156 | 0.876 |
|  | 3 | -.740 | 2.204 | -.335 | .737 |
|  | 4 | -.152 | 1.161 | -.131 | .896 |
|  | 5 | .679 | 1.211 | .561 | .575 |
| *Education level*  *– College* | 2 | -.229 | 1.195 | -.192 | .848 |
|  | 3 | .044 | 2.072 | .021 | .983 |
|  | 4 | .015 | 1.115 | .013 | .990 |
|  | 5 | .632 | 1.165 | .542 | .588 |
| *Education level*  *– Bachelor* | 2 | .463 | 1.234 | .375 | .708 |
|  | 3 | 1.272 | 2.201 | .578 | .563 |
|  | 4 | .831 | 1.159 | .717 | .473 |
|  | 5 | 1.646 | 1.202 | 1.369 | .171 |
| *Education level*  *– Master* | 2 | -.182 | 1.330 | -.137 | .891 |
|  | 3 | .364 | 2.778 | .160 | .873 |
|  | 4 | .488 | 1.251 | .390 | .697 |
|  | 5 | 1.150 | 1.303 | .882 | .378 |
| *Education level*  *– Doctorate* | 2 | -.756 | 1.349 | -.560 | .575 |
|  | 3 | .344 | 2.276 | .151 | .880 |
|  | 4 | -.527 | 1.284 | -.411 | .681 |
|  | 5 | .507 | 1.294 | .392 | .695 |
| *Political identity* | 2 | -.344 | .175 | -1.961 | 0.050 |
|  | 3 | -.234 | .177 | -1.323 | .186 |
|  | 4 | -.429 | .167 | -2.564 | .010 |
|  | 5 | -.368 | .167 | -2.203 | .028 |

*Note.* *β*: Standardized coefficients of multinomial logistic regression

SE: Standard Errors

**References**

Bacon, S. L., Lavoie, K. L., Boyle, J., Stojanovic, J., & Joyal-Desmarais, K. (2021). International assessment of the link between COVID-19 related attitudes, concerns and behaviours in relation to public health policies: optimising policy strategies to improve health, economic and quality of life outcomes (the iCARE Study). BMJ Open, 11(3), e046127. https://doi.org/10.1136/bmjopen-2020-046127

de la Sablonnière, R., Dorfman, A., Pelletier-Dumas, M., Lacourse, É., Lina, J. M., Stolle, D., Taylor, D. M., Benoit, Z., Boulanger, A., Caron-Diotte, M., Mérineau, S., & Nadeau, A. (2020). COVID-19 Canada: The end of the world as we know it? (Technical report No. 1). Presenting the COVID-19 Survey. Université de Montréal.
